# Risk stratification at prediabetes onset and association with diabetes outcomes using EHR data

**DOI:** 10.1038/s44324-025-00091-0

**Published:** 2025-12-17

**Authors:** Junjie Luo, Di Hu, Rui Han, Diyang Lyu, Ritu Agarwal, Nestoras Mathioudakis, Jehan El-Bayoumi, Gordon Gao, Nawar Shara

**Affiliations:** 1 School of Medicine, Johns Hopkins University, Baltimore, MD USA; 2 Donald Bren School of Information and Computer Sciences, University of California Irvine, Irvine, CA USA; 3 Center for Digital Health and AI, Johns Hopkins University, Baltimore, MD USA; 4 MedStar Health, Columbia, MD USA; 5 Georgetown University and MedStar, Washington, DC USA

**Keywords:** Type 1 diabetes, Type 2 diabetes, Pre-diabetes

## Abstract

Prediabetes can progress to type 2 diabetes (T2D), but individual risk varies widely. Few studies have rigorously characterized subgroups at the point of prediabetes (PD) onset. Using electronic health records (EHRs), we developed a machine learning approach to stratify PD and analyze T2D progression risk. We defined PD onset based on strict HbA1c criteria and excluded patients with missing follow-ups or atypical clinical events, yielding a high-fidelity cohort of 14,436 patients from an initial pool of 74,054 (2017–2023, MedStar Health). An XGBoost model using routine features, including HbA1c, BMI, blood pressure, lipids, ALT, medication history, and lifestyle factors, was trained on 2018–2020 data and tested on 2021–2022 patients, achieving an AUC of 81.6%. Risk scores enabled subtyping into high-, medium-, and low-risk groups with distinct progression trajectories. Stratification patterns remained consistent in future cohorts. This approach supports earlier, personalized intervention and diabetes risk prediction using real-world EHR data.

## Introduction

The rising global prevalence of type 2 diabetes (T2D) has become a major public health concern^1^. Prediabetes, characterized by elevated glycemic levels above normal but below the diabetes threshold, represents a significant milestone in the progression towards T2D^2^. Recent statistics show that approximately 98 million American adults, representing more than 1 in 3, have prediabetes^3^. Individuals with prediabetes face a significantly higher risk of developing T2D. Prediabetes onset also presents the opportunity of clinical interventions, such as annual screening, lifestyle programs, and medication, which can delay or prevent the onset of T2D^4,5^.

Patients with prediabetes show varying risks of progressing to diabetes^6^. Personalized risk assessments could optimize resource allocation and facilitate more targeted intervention, which help prevent over-treatment for low-risk patients and under-treatment for high-risk patients, thereby reducing patients’ psychological, financial, and other potential burdens^7–9^. Despite its clinical importance, limited research has systematically explored prediabetes-to-diabetes (PD2D) risk prediction, with only six studies published to date (appendix)^10–19^. Most proposed studies rely on survey program data, which limits the generalizability across healthcare institutions. As a result, few tools are available for clinicians to subgroup prediabetes-onset patient and characterize their progression to diabetes^14–21^.

In this study, we propose to use the standard EHR data that is readily available in most health systems for PD2D risk prediction. However, unlike survey data with standardized patient recruitment and structured follow-ups, EHR-based risk prediction faces two critical challenges^17,18^. The first involves phenotyping to accurately identify prediabetes onset cases, as imprecise identification can contaminate the training dataset. The second involves noisy labeling of outcomes, which is complicated by incomplete clinical records, missing follow-ups, and atypical clinical events. These data quality issues can result in model misalignment, where predictions deviate from intended clinical outcomes.

Our approach detects adult patients at the point of prediabetes onset and labels their one-year diabetes status, while explicitly accounting for missing data and atypical clinical events (e.g., immediate initiation of anti-diabetes treatment). Our machine learning model achieved an AUC of 81.59% on a holdout set of future, unseen patients, with robust and consistent performance across the full range of prediabetes HbA1c levels (5.7–6.4%)^9^. Notably, it identified high-risk patients from the 5.7–5.9% HbA1c group and low-risk patients from the 6.0-6.4% HbA1c group. Based on our AI risk scores, we classify PD onset patients into high-, medium-, and low-risk subgroups. The subgroups serve as stronger predictors for diabetes progression compared to PD onset HbA1c levels. The profiles and progression to diabetes for each subgroup were analyzed. Among patients with PD onset HbA1c level of 5.7–5.9%, which typically was regarded as low risk, 29.9% of patients were classified as medium- or high-risk. In the 6.0-6.4% HbA1c group, which is typically regarded as high risk, 42.6% were classified as low- or medium-risk. Survival analysis confirmed that our risk subgroups significantly distinguish PD onset patients in terms of time to diabetes progression. This highlights the value of our risk subgroups for more precise risk identification.

## Results

### Phenotyping for prediabetes onset patients

**Fig. 1:**
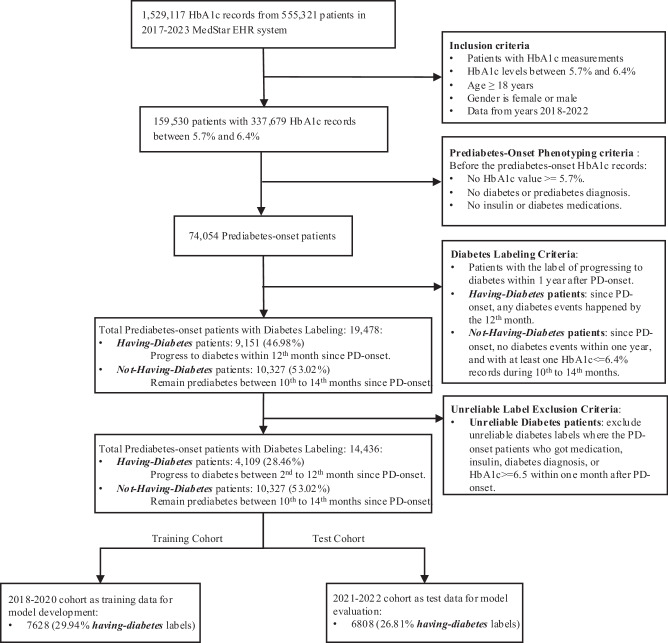
Data collection and cohort segmentation process. This flowchart summarizes the process of identifying prediabetes-onset patients and segmenting cohorts for model development. Starting with 1,529,117 HbA1c records from 555,321 patients (2017–2023) in the MedStar EHR system, inclusion criteria selected 159,530 patients (337,679 records) aged ≥18 years with HbA1c levels between 5.7% and 6.4% (2018–2022). Prediabetes-onset phenotyping yielded 74,054 patients, and our diabetes labeling method classified them as *Having-Diabetes* (9151; 46.98%) or *Not-Having-Diabetes* (10,327; 53.02%) groups. After excluding unreliable labels, 14,436 patients remained (4109 *Having-Diabetes* labels; 28.46%). The dataset was split into a training cohort (2018–2020; 7628 patients, 29.94% *Having-Diabetes*) and a test cohort (2021–2022; 6,808 patients, 26.81% *Having-Diabetes*) for predictive model development and evaluation.

This study identified newly diagnosed adults with prediabetes (PD onset) based on HbA1c measurements in EHR data. As shown in Fig. 1, patients were included if they had a prediabetes-range HbA1c (5.7%–6.4%) record, were over 18 years old, and had no prior diagnosis of diabetes or prediabetes. To ensure we include only new PD onset patients, we require that before the PD onset HbA1c, patients must have had no HbA1c above 5.7%, no diagnosis related to prediabetes or diabetes (Type 1 or Type 2), and no history of glucose-controlling medications use or insulin prescriptions (appendix). We also define *false PD onset* as cases where a patient receives a diabetes diagnosis or treatment within one month after the apparent PD-onset HbA1c record, indicating pre-existing diabetes or off-label early treatment; these are excluded to maintain cohort accuracy. Previous studies failed to accurately identify PD onset patients due to overlooking either historical HbA1c records or medications in their analyses^14–17,19^. These methodological limitations risk misclassifying medically controlled patients with diabetes as new prediabetes cases, which could bias research findings and lead to suboptimal treatment recommendations.

We applied this method to curate the PD2D dataset from the MedStar Health System, an extensive network of clinical facilities in the mid-Atlantic region in the United States, including 10 hospitals and over 300 care locations. The MedStar EHR system contained 1,529,117 HbA1c records from 555,321 patients between 2017 and 2023. Our dataset focuses on the PD onset patients between 2018 and 2022, ensuring a minimum one-year observation period before onset for input data collection and a one-year follow-up period for diabetes status labeling. We identified 337,679 prediabetes-range HbA1c records from 159,530 adult patients. By applying PD onset phenotyping criteria, we established a cohort of 74,054 adult PD onset patients.

### Labeling diabetes status within one-year of prediabetes onset

We use a one-year window after PD onset to evaluate whether a patient has sufficient records to determine their diabetes status. Commonly in clinical practice, some patients did not return for follow-up visits during this period, resulting in incomplete medical records^22^. Previous research typically categorized these patients as *not-having-diabetes* cases by default, which introduces noise. Our study explicitly labeled patients with insufficient data as missing cases and excluded them from training and evaluating diabetes risk scores. Among 74,054 PD onset patients, 54,576 were labeled as missing cases.

Among the remaining 19,478 patients, our method labels patients as having *diabetes* if they meet any of these criteria during the follow-up period: HbA1c ≥ 6.5%, ICD-10 code for T2D, or diabetes medication prescription^23^. Although these criteria are well-known, further review is necessary to determine label uncertainties. First, we excluded Type 1 diabetes patients, as their pathway differs from Type 2. Second, we excluded patients with atypical diabetes-related clinical events that led to *having-diabetes* labels. These cases involved patients labeled *having-diabetes* within one month of PD onset, based on medication (e.g., insulin) or ICD-10 codes, despite HbA1c ≤ 6.5%. This unusually rapid progression suggests these patients were likely already with diabetes but misclassified as PD onset because of incomplete EHRs, or the physician was certain about the high risk of the patient. We want to focus on the PD-onset patients’ progression to diabetes if they are not treated or identified as diabetes within one month. Therefore, we excluded patients labeled *having*-*diabetes* within one month of PD onset to reduce labeling errors^14–17,19^. Among 19,478 PD onset patients, 9151 were initially screened as having *diabetes*. Among them, 62 patients had ICD-10 codes for T1D, and 4980 started medications within one month. They were excluded based on above rationale. The remaining 4109 patients were included as having *diabetes* cases in the training dataset for risk prediction.

To determine *not-having-diabetes* labels, we confirmed there are no diabetes indicators: HbA1c ≥ 6.5%, T2D diagnosis, or diabetes medication prescription. Furthermore, we assessed whether patients remained prediabetes at the end of follow-up. Specifically, we considered HbA1c records < 6.5% between the 10th and 14th months post-onset as evidence of continued prediabetes. Such patients were reassessed at the end of the follow-up period and confirmed to remain prediabetes. Our *not-having-diabetes* definition is more strict than in previous studies, requiring clear EHR evidence of continued non-diabetic status (appendix)^14,15,19^. Among 74,054 PD onset patients, 10,327 remained prediabetes one year after onset.

The next stage subgroup development is based on the 14,436 PD onset patients with reliable future diabetes status in the one-year post window, of whom 4,109 (28.46%) progressed to T2D, and 10,327 (71.54%) remained prediabetes. While the portion of patients progressing to T2D is higher than typical estimate of 5-10%, this is likely due to the incomplete data issue that excludes more patients who are not diabetes.

### Noise-reduced dataset for PD2D task

As shown in Table 1, the final dataset comprises 14,436 PD onset patients with reliable risk labels, systematically categorized by gender, race, age groups, and HbA1C value ranges spanning from 2018 to 2022. Patients were stratified into two ranges based on PD onset HbA1c values: 5.7–5.9% and 6.0–6.4%. In total, 28.46% of patients progressed to diabetes within one year. Gender distribution revealed a higher prevalence in females (8699) compared to males (5737). In terms of race, patients identified as Black or African American constituted the majority of the population, accounting for 6854 individuals, followed by patients identified as White (5549) and those of other races (2033). Regarding age, the 40–59 age group had the highest number of patients (5907).

**Table 1:** Patient Characteristics

	All Patients	Cohort	Prediabetes onset patients with PD-onset HbA1c Group		
		Training Cohort.(2018–2020)	Test Cohort.(2021–2022)	Low PD-onset HbA1c.(5.7–5.9%)	High PD-onset HbA1c (6.0–6.4%)
Total	14,436 (28.46%)	7628 (29.94%)	6808 (26.81%)	9449 (18.52%)	4987 (47.30%)
Black	6854 (30.51%)	3971 (30.57%)	2883 (30.42%)	4217 (20.87%)	2637 (45.92%)
Other	2033 (24.89%)	874 (26.43%)	1159 (23.73%)	1372 (15.60%)	661 (44.18%)
White	5549 (27.25%)	2783 (30.15%)	2766 (24.33%)	3860 (16.99%)	1689 (50.68%)
Female	8699 (27.68%)	4492 (28.78%)	4207 (26.50%)	5810 (18.42%)	2889 (46.31%)
Male	5737 (29.65%)	3136 (31.60%)	2601 (27.30%)	3639 (18.69%)	2098 (48.67%)
18-39	1967 (31.52%)	910 (30.22%)	1057 (32.64%)	1479 (23.94%)	488 (54.51%)
40-59	5907 (23.75%)	3157 (23.88%)	2750 (23.60%)	3997 (15.26%)	1910 (41.52%)
60-74	4682 (29.24%)	2571 (31.82%)	2111 (26.10%)	2893 (17.49%)	1789 (48.24%)
75+	1880 (38.14%)	990 (44.14%)	890 (31.46%)	1080 (25.93%)	800 (54.62%)

This table displays patient counts by race, gender, and age groups, with data split between Training (2018–2020) and Test (2021–2022) cohorts, and stratified by HbA1c ranges (5.7–5.9% and 6.0–6.4%).

All data includes percentages shown in parentheses, indicating the number of PD-onset patients and progression to diabetes rate within one year.

### Model performance for PD2D risk score

**Fig. 2:**
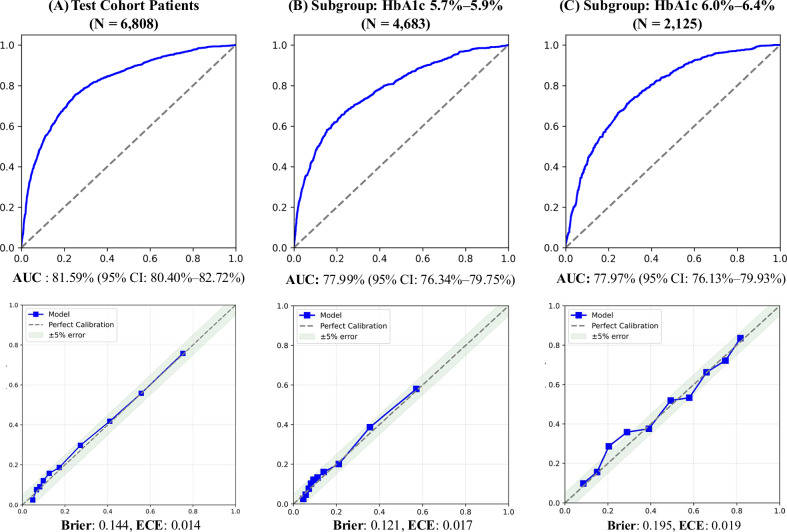
ROC and calibration curve of the PD2D risk model across HbA1c subgroups. Receiver Operating Characteristic (ROC) and calibration curves are shown for the PD2D risk model in (**A**) the full test cohort, (**B**) the subgroup with HbA1c 5.7%–5.9%, and (**C**) the subgroup with HbA1c 6.0%–6.4%. ROC curves (top row) demonstrate strong discrimination in identifying high-risk patients, with AUCs of 81.59% (95% CI: 80.40%–82.72%), 77.99% (95% CI: 76.34%–79.75%), and 77.97% (95% CI: 76.13%–79.93%), respectively. Calibration curves (bottom row) indicate close agreement between predicted and observed diabetes progression probabilities, with Brier scores of 0.144, 0.121, and 0.195, and expected calibration errors (ECE) of 0.014, 0.017, and 0.019. Together, these results demonstrate that the model maintains both strong discrimination and good calibration across HbA1c strata in the test cohort.

The model demonstrates robust performance among the unseen future patients (2021-2022), as shown in both Table 2 and Fig. 2. The overall AUC is 81.59%, showing the performance for distinguishing PD onset patients, whose condition progressed to diabetes within one year or not. Testing on future unseen patients, our model still maintains high predictive performance. As such, the model’s predictions can be confidently used for identifying patients at higher risk of developing diabetes, thereby supporting proactive healthcare interventions. Moreover, to assess whether the model is primarily driven by the PD onset HbA1c, we examined its performance across different HbA1c ranges. AUC scores remain high: 77.99% for patients with the 5.7–5.9% PD onset HbA1c range and 77.97% for patients with higher PD onset HbA1c levels (6.0–6.4%). This indicates the prediction power is not driven solely by baseline HbA1c levels but also by other EHR information.

**Table 2:** Model Performance Comparison Across Different HbA1C values in Prediabetes-Onset

Name	Sample Num	Diabetes Rate within One Year	Method 1: Ranking with PD-onset HbA1c			Method 2: Ranking with Our AI Risk Score			
			AUROC	Diabetes Rate of Patients Among Top 10%	Diabetes Rate of Patient Among Bottom 10%	AUROC	Diabetes Rate of Patients Among AI Risk Score > 0.5	Diabetes Rate of Patients Among Top 10%	Diabetes Rate of Patient Among Bottom 10%
Test Cohort (2021–2022)	6808	26.81%	68.70% (67.25%-70.06%)	62.65%	14.81%	81.59% (80.40%–82.72%)	60.64%	76.47%	2.93%
Test Cohort,PD-onset HbA1c in range of 5.7–5.9%	4683	18.34%	54.80% (52.85%–56.88%)	18.38%	14.04%	77.99% (76.34%–79.75%)	54.33%	61.32%	2.77%
Test Cohort, PD-onset HbA1c in range of 6.0–6.4%	2125	45.46%	67.34% (65.30%–69.61%)	72.64%	22.90%	77.97% (76.13%–79.93%)	64.32%	82.08%	10.75%

Table 2 presents sample size, and one-year diabetes progression rates, and model performances for different HbA1c subgroups (5.7–5.9% and 6.0–6.4%). The table compares two predictive models (PD-onset HbA1c and AI Risk Score) using metrics including AUC, accuracy, and precision across different HbA1c ranges in the Test 2021–2022 cohort.

To evaluate model calibration, we computed the Brier score and expected calibration error (ECE) and plotted calibration curves for the test cohort and two HbA1c subgroups (Fig. 2). The model demonstrated good calibration, with Brier = 0.144 and ECE = 0.014 in the overall test cohort. Calibration remained stable across both HbA1c subgroups (5.7–5.9%: Brier = 0.121, ECE = 0.017; 6.0–6.4%: Brier = 0.195, ECE = 0.019). These results indicate that predicted probabilities closely matched observed diabetes progression rates, supporting the model’s reliability for risk communication and clinical decision-making.

Table 2 also shows our AI score ranking outperforms the PD onset HbA1c values in terms of identifying high-risk PD onset patients. Method 1 ranks patients based solely on their PD onset HbA1c levels, while Method 2 uses our AI risk scores to rank the PD onset patients. As shown in the table, Method 2 consistently outperforms Method 1 across all metrics. Specifically, Method 2 achieves higher AUROC scores (81.59% overall) compared to Method 1 (74.18% overall), indicating better predictive performance. Additionally, Method 2 identifies a greater proportion of high-risk patients among the top 10% (76.47% vs. 62.65%) and significantly reduces the misclassification of low-risk patients as high-risk among the bottom 10% (2.93% vs. 14.81%).

Notably, our method also shows robust performance across different HbA1c subgroups. Patients with lower PD onset HbA1c values may be at high risk of progressing to diabetes, while patients with higher HbA1c levels may exhibit low risk. In the test cohort, for patients with 5.7–5.9% PD onset HbA1c, the progression to diabetes rate within one year is 18.34%. While for patients with 10% highest risk score, their diabetes rate is 60.26%. For the patients of 6.0–6.4% PD onset HbA1c, the diabetes rate is 45.46%. While for patients with the 10% lowest risk scores, their diabetes rate is 11.21%. This finding suggests that HbA1c levels alone may not fully capture a patient’s progression risk, highlighting the importance of using a more holistic approach to identify patients who are likely to progress to diabetes within one year.

### Profiling analysis for PD-onset subgroups

The subgroup analysis involved stratifying patients into risk subgroups based on calculated AI risk scores. Our stratification approach utilized a normalized scale (0-1) to define the PD onset subgroups: patients with scores exceeding 0.8 were high-risk, below 0.2 were low-risk, and others were medium-risk. Then we examined the association between these subgroups’ clinical profile patterns and diabetes-related clinical outcomes.

**Fig. 3:**
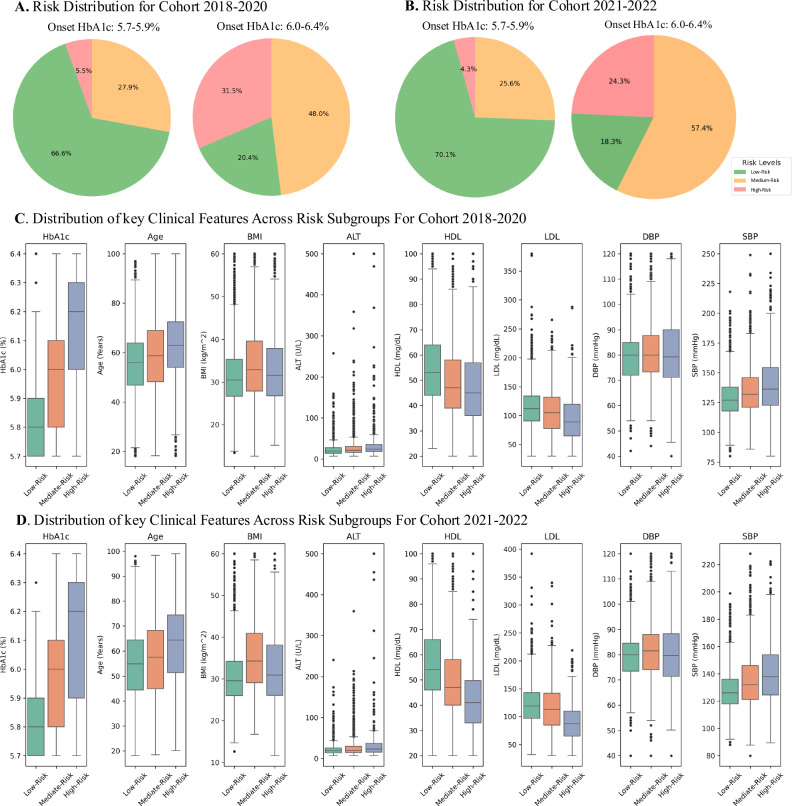
Statistics descriptions for prediabetes-onset patients from low, medium, or high-risk subgroups. **A** Distribution of risk levels in training cohort (2018-2020) patients by HbA1c ranges (5.7–5.9%: *n* = 4766; 6.0-6.4%: *n* = 2862). **B** Distribution of risk levels in testing cohort (2021–2022) patients by HbA1c ranges (5.7–5.9%: *n* = 4683; 6.0–6.4%: *n* = 2125). **C** Distribution of clinical features (HbA1c, Age, BMI, ALT, HDL, LDL, DBP, SBP) across three risk levels (low-risk, middle-risk, high-risk) in the 2018–2020 training cohort. **D** Distribution of the same clinical features across risk levels in the 2021–2022 testing cohort. Box plots show median, interquartile range, and outliers for each parameter.

Figure 3A, B represent the risk distribution across different PD onset HbA1c strata: 5.7–5.9% and 6.0–6.4% for both training (2018–2020) and test (2021–2022) cohorts. In the 5.7–5.9% group, only around two-thirds are low risk (67.0% in training cohort and 70.2% in the test cohort), indicating that even individuals with lower HbA1c values may be at substantial risk of developing diabetes. As HbA1c levels increase, the percentage of high-risk individuals rises. In the 6.0–6.4% group, nearly 32.3% of the 2018–2020 cohort and 23.7% of the 2021–2022 cohort are high-risk subgroups, reflecting a clear correlation between elevated HbA1c and increased diabetes risk.

Figure 3C, D compare distributions of key clinical factors across three risk-subgroups for both training and test cohorts. Each box plot provides a visual summary of how clinical characteristics such as HbA1c, age, BMI, ALT, HDL, LDL, DBP, and SBP vary by risk category. Analysis revealed significant associations between diabetes risk stratification and multiple clinical parameters, aligning with established literature findings. HbA1c, age, and BMI exhibited a marked elevation in higher risk categories. ALT levels demonstrated substantial variability across all risk groups, with numerous high-value outliers, particularly in the middle and high-risk categories. Cardiovascular parameters showed distinct patterns: HDL displayed a negative association with risk (decreasing from low to high-risk), while LDL showed less pronounced differences between groups but maintained slightly elevated levels in higher risk categories. Blood pressure measurements revealed a positive association with risk level, with both DBP and SBP showing progressive increases across risk categories. These observed relationships are mechanistically supported by extensive research on insulin resistance, systemic inflammation, and metabolic syndrome pathophysiology^24,25^. The box plots also reveal considerable overlaps between risk categories for most parameters, suggesting the complex, multifactorial nature of diabetes risk assessment. Also, the consistency in the distribution of these clinical features between the two cohorts reflects the robustness of the risk stratification model.

### Diabetes outcome analysis for PD-onset subgroups

**Fig. 4:**
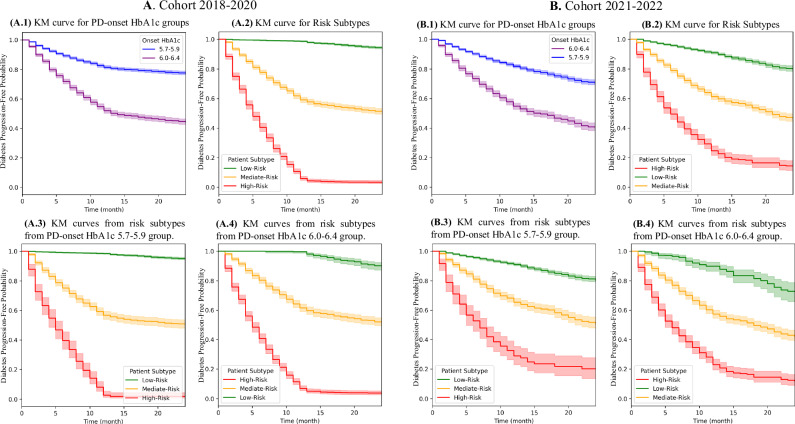
Kaplan-Meier survival curves for progression to diabetes among prediabetes-onset patients. **A.1** KM curve stratified by HbA1c levels for cohort 2018–2020. **A.2** KM curve stratified by risk subgroups for cohort 2018–2020. **A.3**–**4** KM curve stratified by risk subgroups within HbA1c 5.7–5.9% and 6.0–6.4% groups for cohort 2018–2020. Shaded areas: 95% confidence intervals. **B.1** KM curve stratified by HbA1c levels for cohort 2021–2022. **B.2** KM curve stratified by risk subgroups for cohort 2021–2022. **B.3**–**4** KM curve stratified by risk subgroups within HbA1c 5.7–5.9% and 6.0–6.4% groups for cohort 2021–2022. Shaded areas: 95% confidence intervals.

The Kaplan-Meier survival curves presented in Fig. 4 illustrate the progression of PD2D, categorized by their PD onset HbA1c levels, from two distinct cohorts: 2018–2020 and 2021–2022 and two subgroups: 5.7–5.9%, and 6.0–6.4%. The survival probability, defined as the likelihood of remaining free from diabetes, decreases over time across all groups. However, patients with higher onset HbA1c levels (6.0–6.4%) demonstrate a more rapid decline in survival probability, indicating a faster progression towards diabetes compared to those with lower onset HbA1c levels. This trend is consistent across both cohorts, even though they are from two different periods. It reflects the inherent characteristics of the PD onset patients.

Figure 4 presents Kaplan-Meier survival curves demonstrating the progression to diabetes over time for three patient subgroups (low-risk, medium-risk, and high-risk) grouped by the risk scores. The left panel shows data from the training (2018–2020) cohort, while the right panel illustrates data from the test (2021–2022) cohort. The survival curves reveal distinct patterns of diabetes progression among the three subgroups. Patients in the high-risk group (depicted in red) exhibit a steep decline in diabetes progression-free probability, with a large proportion progressing to diabetes within the first few quarters of follow-up. In contrast, medium-risk patients (shown in yellow) display a more gradual decline in survival probability, indicating a medium rate of progressing to diabetes. Low-risk patients (depicted in green) maintain a consistently high survival probability throughout the observation period, reflecting a significantly reduced likelihood of PD2D progression.

The consistency of survival patterns between the training (2018–2020) and test (2021–2022) cohorts highlights the robustness of the risk stratification model. Despite differences in the time periods represented, the model reliably identifies patient subgroups with similar progression trajectories. This suggests that the system remains effective in classifying patients and predicting diabetes outcomes in future, unseen populations. The consistency across both cohorts supports the generalizability of the model for clinical application in identifying patients at varying levels of risk for diabetes progression.

## Discussion

Our study built an EHR dataset with more precise labeling of prediabetes onset and diabetes status. Using data from a large U.S. health system, we developed a diabetes risk score model that predicts an individual’s personalized risk of progressing to diabetes within one year. The model demonstrated high accuracy and robustness when being evaluated on future unseen patients. Subsequently, our analysis confirmed the associations between PD onset patient risk subgroups and progress to diabetes.

First, we highlighted two key challenges in creating a high-quality PD2D dataset: accurately identifying prediabetes onset patients and establishing reliable labels. Our study tackles these challenges through rigorous phenotyping rules that incorporate historical HbA1c levels, medication records, and diagnosis data, along with stringent risk labeling criteria that consider both EHR data absence and atypical diabetes-related clinical events.

Second, based on our curated dataset, we developed a precise risk scoring system achieving an AUC score of 81.59%, with replicability for future unseen patients. Besides, the model exhibited good calibration with Brier Score of 0.144, supporting its clinical interpretability for risk communication and potential threshold-based interventions. Our model demonstrates its ability to identify high-risk patients even within lower PD onset HbA1c ranges (5.7–5.9%), as well as low-risk patients within higher HbA1c ranges (6.0–6.4%). This suggests that our risk assessment approach serves as a more reliable indicator of diabetes progression than relying solely on prediabetes onset HbA1c levels.

Third, we conducted a comprehensive analysis of patient subgroups and revealed clear associations between clinical parameters and diabetes progression risk, offering valuable insights for clinical practice. Additionally, the stratification of patients into distinct risk groups effectively predicted their progression to diabetes, as evidenced by the significantly different survival trajectories in the Kaplan-Meier analysis. The consistency of these patterns across different time periods and patient populations validates the reliability of our risk stratification model and suggests its potential utility as a clinical tool for assisting early interventions. Our results show that nearly one-third of patients with PD onset HbA1c levels between 5.7% and 5.9%, typically classified as low risk in standard screening, were identified as medium or high risk by our model. This finding points to a potential gap in current prediabetes management guidelines. In practice, reclassifying these patients as higher risk could encourage more frequent follow-up visits, structured lifestyle counseling, or additional metabolic monitoring in primary care. Such a risk-informed strategy may enable earlier and more personalized preventive actions. Its predictive capability could help identify high-risk prediabetes patients for timely targeted interventions and personalized prevention.

Several limitations warrant consideration in this study. First, the generalizability of our diabetes risk model may be limited due to its development using a subset of the PD-onset population with available labels: only 19.49% (14,436/74,054) of PD onset patients have reliable labels for model development. Second, the retrospective analysis of EHR data necessitated the exclusion of patients with missing outcome information, potentially introducing selection bias as patients with complete follow-up data may systematically differ from those without. Third, our definition of diabetes onset is based on a single abnormal HbA1c value, whereas clinical diagnosis typically requires two abnormal results or a random glucose ≥200 mg/dL with symptoms. Future research should validate these findings across diverse populations and address potential selection biases.

In conclusion, this study contributes to PD2D research through three key advancements: a novel approach for constructing less biased EHR-based PD2D datasets, a precise predictive model that identifies PD2D risk at the individual level across HbA1c levels, and a comprehensive patient subgroup analysis that enhances our understanding of clinical parameters and PD2D progression. These findings contribute to advancements in both the methodology of prediabetes research and its clinical applications.

## Method

### Study design and data source

This retrospective cohort study used de-identified electronic health records (EHRs) from the MedStar Health System covering January 2017 through December 2023. Data were drawn from ten hospitals and over 300 outpatient facilities in the U.S. mid-Atlantic region. The Johns Hopkins School of Medicine Institutional Review Board approved the protocol (IRB00411858) with a waiver of informed consent owing to the use of anonymized, non-interventional data. All TRIPOD items relating to data source and ethical approval are fully addressed.

### Participants and eligibility criteria

We identified our study population by querying the MedStar Health System’s EHR for adults (age ≥ 18 years) whose first-ever HbA1c measurement fell within the prediabetic range of 5.7%–6.4% between 2018 and 2022. This “prediabetes onset” date marks the index event for each patient and serves to capture individuals newly confronting impaired glucose regulation, without any prior clinical indication of diabetes status or exposure to diabetes-related care. By anchoring cohort entry to this specific laboratory value, we ensure that our analyses focus exclusively on incident prediabetes cases rather than on patients with established disease or prior management.

To verify true incident prediabetes, we conducted a comprehensive review of each patient’s longitudinal record, extending up to ten years prior to the index HbA1c to exclude any evidence of earlier glycemic impairment or treatment. Patients were required to have no previous HbA1c reading in the prediabetic or diabetic range ( ≥ 5.7%), no diagnostic codes for prediabetes or for Type 1 or Type 2 diabetes, and no prescriptions for glucose-modulating medications or insulin prior to their index measurement. This rigorous, systematic phenotyping approach minimizes misclassification by screening out individuals who may have achieved a prediabetic-range HbA1c through prior therapy or whose diabetes status was already clinically recognized.

By combining a precise laboratory-based definition with exhaustive historical screening, our eligibility criteria isolate a clean cohort of adults at genuine first onset of prediabetes. This method enhances internal validity, allowing us to map early progression trajectories and to identify optimal windows for preventive interventions unfettered by confounding from prior glycemic management or patient education. A detailed comparison of existing literature approaches and our systematic phenotyping methodology is provided in the Appendix.

### Outcome definition

The primary goal of our prediabetes-to-diabetes (PD2D) prediction task is to infer each patient’s true progression risk (high, low, or misclassified) based on longitudinal EHR data collected after their prediabetes onset. Because true future diabetes status is not directly observable at the time of the index HbA1c measurement, we operationalize ground truth by extracting all relevant clinical events over the subsequent 12-month horizon. The timestamp of the first HbA1c record in the 5.7%–6.4% range defines the “prediabetes onset” datetime, at which point historical data are frozen and model features assembled. We then assemble “future EHRs,” encompassing laboratory values, diagnosis codes, and medication records from that index point forward.

Within the future EHR window, we distinguish diabetes events, defined as any HbA1c ≥ 6.5%, any ICD-10 code for Type 1 or Type 2 diabetes, or the initiation of anti-diabetic therapy, from non-diabetic events (predominantly HbA1c < 6.5%). Patients are labeled as *having-diabetes* if they experience one or more diabetes events within one year of prediabetes onset. Those who maintain HbA1c values below the diagnostic threshold and do not receive diabetes ICD-10 codes or prescriptions during the entire follow-up period are classified as *not-having-diabetes*. As defined earlier, cases in which anti-diabetic treatment occurs within one month of the index HbA1c are treated as false PD onset and excluded, since such early interventions likely reflect pre-existing but undocumented diabetes or off-label prediabetes treatments. For the selected PD-onset patients with off-label prediabetes treatment, we exclude them as their nature progression to diabetes are influenced.

Moreover, different from the previous study, we further refine our *not-having-diabetes* definitions with a time-aware approach. Instead of equally counting any normal HbA1c reading within one year since PD-onset, we focus on HbA1c < 6.5% records occurring near the end of the 12-month horizon (months 10–14) to label “*not-having-diabetes*”, as these readings better reflect a patient’s status at the prediction target. Patients lacking any follow-up records are not automatically deemed low-risk; instead, we assign a “missing label” to avoid conflating loss to follow-up with true non-progression.

By integrating both diabetes and non-diabetic events, excluding atypical or off-label early interventions, and weighting evidence by its temporal proximity to the one-year mark, our outcome definition aims to create precise, clinically meaningful labels for PD2D risk. A detailed comparison of our labeling methodology against existing approaches from the literature, diabetes event definition, ICD-10 list, and anti-diabetes medication list are provided in the Appendix.

### Predictor variables

We derived our predictor variables from structured EHR data observed during the one-year period preceding each patient’s prediabetes onset date. To capture both the patient’s underlying physiology and patterns of healthcare utilization, we organized features into three broad domains: laboratory and vital signs, lifestyle behaviors, and medical/diagnostic information. For each domain, we computed both aggregated mean values and record counts.

Laboratory and vital sign features included baseline measures of glycemic control (HbA1c), body mass index (BMI), liver function (ALT), lipid profiles (HDL, LDL), and blood pressure (systolic and diastolic)^14,15,19^. For each of these numeric variables, we calculated the mean value over the look-back period to obtain a stable indicator of central tendency and the total number of observations to reflect monitoring intensity. Lifestyle features include alcohol consumption, exercise frequency, diet patterns, and smoking status^26^. They are similarly summarized as the mean categorical feature frequency and the number of encounters. Medical and diagnostic features encompassed ordered medications and ICD diagnosis codes; we represented diagnoses both at the full code level and via three-character prefixes to allow analysis at differing levels of clinical granularity, and again captured both average feature frequency and total record count.

Missing data are inevitable in real-world EHRs. For continuous lab and vital sign values without any recorded measurement, we imputed the median value calculated from the training cohort and introduced a binary “missingness” indicator flag to preserve information about data absence. Categorical lifestyle and diagnostic features with no entries during the look-back were assigned an “unknown” feature category. Count features defaulted to zero when no records existed, naturally distinguishing patients with no documented encounters from those with sparse but non-zero activity. This combined strategy of cohort-based imputation, missingness indicators, and zero-fill counts ensures robust model training while retaining the signal embedded in patterns of missingness. Details of feature engineering are provided in the appendix.

### Model building and evaluation

The diabetes risk-rating system was developed using XGBoost, a gradient boosting framework that utilizes decision tree ensembles^27^. It is an efficient gradient-boosted tree ensemble algorithm chosen for its capacity to handle high-dimensional, sparse EHR feature vectors and capture complex, non-linear associations. Patients with prediabetes onset between 2018 and 2020 comprised the training cohort, while those from 2021 and 2022 formed an independent testing cohort to assess temporal generalizability. Within each cohort, we further stratified patients by their index HbA1c (5.7%–5.9% vs. 6.0%–6.4%) to examine performance across differing baseline risk levels. All input features, including aggregated means and encounter counts for laboratory values, vitals, lifestyle factors, and diagnostic/medication records, were preprocessed into sparse matrices using the scikit-learn API. To optimize predictive performance while minimizing the risk of overfitting, we employed a Bayesian optimization approach supplemented by grid search refinement for hyperparameter tuning. The key parameters adjusted included the number of boosting iterations (n_estimators), the maximum depth of each tree (max_depth), the learning rate (eta), and the early stopping criteria. Candidate configurations were evaluated using five-fold cross-validation on the training dataset, with log loss serving as the optimization objective. The final model was trained using the optimal hyperparameter values, which specified a maximum tree depth of ten, one thousand boosting iterations, a learning rate of 0.01, and a binary logistic objective function.

Model performance was assessed with multiple complementary metrics. Discrimination was measured by the area under the receiver-operating characteristic curve (AUC), with empirical 95% confidence intervals derived from 2000 stratified bootstrap replicates. To evaluate classification accuracy, we applied clinically relevant thresholds: an HbA1c cutoff of 6.0% and a risk-score threshold of 0.5, and then calculated the overall proportion of correct predictions. Recognizing the importance of identifying both the highest- and lowest-risk patients, we also report precision within the top and bottom deciles of predicted risk scores. Precision at the top 10% quantifies our ability to capture true progressors among those deemed highest risk, while precision at the bottom 10% reflects specificity in identifying patients unlikely to progress. This multifaceted evaluation framework ensures that our model is robust, clinically interpretable, and directly aligned with decision thresholds used in practice.

### Risk subgroup profiling and future diabetes progression analysis

Previous PD2D studies have provided valuable tools for predicting diabetes risk; however, opportunities remain to explore clinical profiles and progression patterns within different risk-based subgroups. Understanding these trajectories is essential for personalizing prediabetes management beyond one-size-fits-all approaches. Building on the framework demonstrating how distinct patient profiles affect disease progression in diabetes-onset subgroups, we extended this concept to prediabetes populations^28^. By examining risk subgroups based on clinical profiles, we sought to identify patient clusters characterized by unique progression patterns and rates, potentially enabling targeted preventive strategies.

In this study, patients were classified into high-, medium-, and low-risk subgroups using risk score thresholds of 0.8 and 0.2, respectively. We conducted a comprehensive analysis of these subgroups, focusing on three key dimensions: (1) the association between risk subgroups and prediabetes onset HbA1c levels, visualized through pie charts; (2) the distribution of key electronic health record (EHR) measures across risk subgroups; and (3) diabetes progression patterns specific to each risk subgroup. To validate the generalizability of our findings, we performed parallel analyses on both training and testing cohorts, assessing the replicability of subgroup characteristics in previously unseen patients. Beyond improving clinical decision-making, this subgroup analysis provides critical insights into mechanisms driving diabetes progression. Identifying how specific clinical factors contribute to differential risk profiles refines our understanding of disease development, potentially enhancing early intervention efforts and optimizing resource allocation. Furthermore, tracking progression to diabetes within these subgroups serves as a valuable real-world validation of the methodology, creating an iterative feedback loop where observed outcomes can refine future subgroup definitions and risk assessment approaches.

### Statistical analysis

For patient-level descriptive statistics, categorical variables were reported as absolute and relative frequencies, while quantitative variables were presented as mean (standard deviation). All analyses were conducted in Python (version 3.11.18) using pandas, matplotlib, plotly, XGBoost, sklearn, and lifelines packages. For subgroup development, we implemented gradient boosting using the xgboost package. Receiver operating characteristic (ROC) curves were employed to identify risk factor thresholds for diabetes risk prediction^29^. The testing dataset was further stratified into groups with HbA1c levels of 5.7–5.9% and 6.0–6.4% at PD onset to evaluate model performance. For subgroup analysis, Kaplan-Meier survival curves were used to analyze the patients’ progression to diabetes^30^.

## Supplementary information

PD2D-Appendix-1105.

## Data Availability

This study used de-identified electronic health records (EHRs) from the MedStar Health System. Due to institutional and patient privacy regulations, the raw EHR data cannot be made publicly available. Researchers interested in accessing the dataset for replication or collaborative research may contact the corresponding author, subject to data use agreements and IRB approval.

## Code availability

The code used for data preprocessing, risk score modeling, and subgroup analysis is available on GitHub at: https://github.com/JHU-CDHAI/1stPD2D.
