# Supplementary material for: Risk stratification at prediabetes onset and association with diabetes outcomes using EHR data
Source: NPJ Metab Health Dis. 2025 Dec 17;3:48. doi: 10.1038/s44324-025-00091-0 (PMC12711876; doi:10.1038/s44324-025-00091-0)
Supplement: Supplementary file 1 — PD2D-Appendix-1105. [file 44324_2025_91_MOESM1_ESM.pdf]

Supplementary Appendix

Title:

Risk Stratification at Prediabetes Onset and Association with Diabetes Outcomes Using EHR Data

Table of Contents

Risk Stratification at Prediabetes Onset and Association with Diabetes Outcomes Using EHR Data 1

1. Related Research 3

1.1 Data Source for the Prediabetes-to-Diabetes (PD2D) Task 3

1.2 Phenotyping Rules for Prediabetes onset Patients 4

1.3 Diabetes Labeling Method 5

1.4 Patient Subgroup Analysis 7

2. Diabetes and None-Diabetes Events 9

2.1 Definitions of Diabetes and Non-diabetes Events 9

2.2 Diabetes Medication List 9

2.3 Diagnosis List 11

3. Input EHR Features 13

4. Model Development and Evaluation 16

5. Patient Subgroup Description Table 18

6. KM Survival Table for PD onset HbA1C subgroups 20

6.1 2018-2020 Cohort across Prediabetes onset HbA1c. 20

6.2 2021-2022 Cohort across Prediabetes onset HbA1c. 22

7. KM Survival Table for PD onset HbA1C subgroups 24

7.1 2018-2020 Cohort across Risk Subgroups. 24

7.2 2021-2022 Cohort across Risk Subgroups. 27

8. KM-Survival Tables for PD onset HbA1c group's Risk Subgroups. 30

8.1 2018-2020 Cohort with 5.7-5.9 PD onset HbA1c across Risk Subgroups. 30

8.2. 2018-2020 Cohort with 6.0-6.4 PD onset HbA1c across Risk Subgroups. 33

8.3. 2021-2022 Cohort with 5.7-5.9 PD onset HbA1c across Risk Subgroups. 36

8.4. 2021-2022 Cohort with 6.0-6.4 PD onset HbA1c across Risk Subgroups. 39

Reference 42

1. Related Research

This section provides a comprehensive review of existing research on predicting the progression from prediabetes to diabetes (PD2D), highlighting methodological approaches and identifying critical gaps that our study addresses. We examined four key areas: the data sources used in previous studies, phenotyping approaches for identifying prediabetes onset patients, methods for labeling diabetes progression, and the need for patient subgroup analysis.

Our research advances the field by leveraging extensive electronic health records (EHRs) from a major health system in the U.S. that captures patient data across multiple care settings. While many foundational studies have relied on health surveys or laboratory networks that provide valuable cross-sectional data points, our approach complements this body of work by utilizing continuous, longitudinal clinical data to develop more robust progression models. We introduce rigorous phenotyping rules to accurately identify prediabetes onset patients, implement comprehensive labeling methods that consider both diabetes and non-diabetes events over time, including the atypical diabetes events that happened immediately after prediabetes onset, and conduct detailed subgroup analyses to understand distinct clinical patterns and progression trajectories.

1.1 Data Source for the Prediabetes-to-Diabetes (PD2D) Task

Previous studies on predicting the progression from prediabetes to diabetes (PD2D) have used diverse data sources, each offering distinct strengths and extra space to improve for modeling disease trajectories.

National Health Surveys and Screening Programs
Several influential studies have leveraged population-based data collections. Nicolaisen et al.[1] utilized the Danish National Health Survey, linking it to nationwide registries, incorporating laboratory results, socioeconomic indicators, and self-reported lifestyle factors to develop population-level predictive models. In China, Liu et al.[2] analyzed data from a Wuhan health screening program targeting adults aged 65+, capturing detailed metabolic parameters, including fasting plasma glucose and anthropometric measurements. Similarly, Han et al.[3] examined records from over 211,000 participants in the Rich Healthcare Group's check-up program (2010-2016), identifying key diabetes risk factors based on demographic, lifestyle, and laboratory information. In the United States, Aoki et al.[4] analyzed an outpatient laboratory network dataset, focusing specifically on longitudinal HbA1c and serum glucose trends over five years to improve biochemical risk identification.

Electronic Health Records (EHRs)
While survey or screening data provide valuable snapshots of health information at specific time points, EHRs offer a complementary perspective by capturing continuous, longitudinal

documentation of patient interactions with healthcare systems. To date, two notable studies have specifically leveraged EHR data for PD2D prediction. Cahn et al.[5] analyzed the THIN database, a UK primary care dataset containing records for over 16 million individuals, integrating laboratory results, demographics, and medication histories, while validating their models using external datasets from Canada and Israel. Zueger et al.[6] examined EHRs from an Israeli health provider (2003-2013), combining biomarkers, demographic data, and disease codes to predict progression risk. These studies highlight the potential of EHRs to support rich modeling of disease trajectories.

Our Approach

Building on these foundational efforts, our study utilizes EHR data from a comprehensive U.S. healthcare system that integrates information across inpatient, outpatient, and emergency department settings. This approach extends prior EHR-based PD2D research in several meaningful ways: (1) whereas Cahn et al.'s study focused on primary care records, our dataset captures the full spectrum of care environments[5]; (2) our data reflect more recent clinical practice, covering the recent past five years and providing updated clinical insights beyond the earlier 2003-2013 window examined by Zueger et al.[6]; and (3) our U.S.-based healthcare system data contributes geographical diversity to the predominantly European and Asian datasets in existing literature. In doing so, we aim to contribute to a contemporary and comprehensive perspective to PD2D prediction research.

1.2 Phenotyping Rules for Prediabetes onset Patients

Patient Population Focus

In this study, we focus on adults experiencing prediabetes onset (PD onset), defined as the initial clinical presentation of prediabetes in previously normoglycemic individuals. These PD onset patients represent a distinct cohort characterized by the absence of prior diabetic or prediabetic indicators. We hypothesize that these individuals have had no prior exposure to diabetes-related health information and are encountering prediabetes management for the first time. Risk stratification among PD onset patients holds particular significance for early intervention and prevention strategies.

Limitations in Previous Approaches

Among existing PD2D studies, only Nicolaisen et al.[1] specifically examined prediabetes onset patients using National Survey data, while other research primarily focused on established prediabetes patients. This distinction is crucial, as patients with established prediabetes may already be undergoing treatment or have awareness of diabetes management, potentially introducing confounding factors into predictive modeling. The development of precise phenotyping criteria is essential for accurately identifying true PD onset patients, as including individuals with normal glycemic status or pre-existing diabetes could introduce significant bias and undermine model validity.

Previous EHR-based studies employed a variety of thoughtful strategies, each reflecting different operational definitions of prediabetes. Cahn et al.[5] established prediabetes criteria as either a glucose value ≥100 mg/dL or HbA1c ≥5.7%, while excluding patients on glucose-lowering medications or meeting diabetes criteria. One consideration in this approach is its reliance on glucose values, which exhibit considerable temporal variability compared to HbA1c measurements. Similarly, Zueger et al.[6] defined prediabetes through either HbA1c values between 5.7% and 6.4% or relevant ICD-9 codes. While effective for large-scale identification, this strategy might not fully distinguish previously diagnosed T2D patients who had achieved prediabetic-range HbA1c through management, potentially compromising internal validity.

Our Systematic Phenotyping Approach

To address these limitations, we present a comprehensive, systematic phenotyping methodology for accurately identifying PD onset patients. Our approach begins by identifying each HbA1c value within the prediabetes range (5.7-6.4%) as a candidate PD onset point. For each candidate, we retrieve the patient's complete historical EHR data spanning up to 10 years before this measurement. We then systematically screen these historical records for any diabetes- or prediabetes-related events to determine whether this point represents genuine prediabetes onset. Our inclusion criteria require:

1. Documented prediabetic-range (PD-range) HbA1c (5.7%-6.4%). We treat the first recorded PD-range HbA1c as the potential PD onset HbA1c.
2. Age ≥18 years
3. Absence of prior diabetic or prediabetic documentation before PD onset HbA1c, specifically:
   - No previous HbA1c readings ≥5.7%
   - No diagnostic codes related to prediabetes or diabetes (Type 1 or Type 2)
   - No history of glucose-modulating medication or insulin prescriptions

This structured and longitudinal approach ensures the accurate identification of incident prediabetes cases, enhancing our understanding of early diabetes progression trajectories and facilitating the identification of potential therapeutic intervention windows. By focusing specifically on PD onset patients and implementing refined phenotyping criteria, our study addresses a critical gap in existing research and provides a foundation for developing more targeted early intervention strategies.

1.3 Diabetes Labeling Method

The prediabetes to diabetes (PD2D) prediction task aims to forecast patients' progression from prediabetes to diabetes after their prediabetes onset. This task presents a fundamental challenge: patients’ actual risk level (whether high or low, as ground truth) is not directly observable at the

time of prediabetes diagnosis. To address this, we track future EHRs data to identify relevant clinical events that serve as indicators of the patient's underlying ground truth of patient risk.

Ground Truth Categories and Key Definitions

After applying our phenotyping rules to identify PD onset patients, each patient falls into one of three possible ground truth categories: (1) true PD onset with high risk (progressing to diabetes within one year), (2) true PD onset with low risk (maintaining prediabetic status for at least one year), or (3) false PD onset cases (patients who were actually diabetic when identified as PD onset). These ground truth categories can only be determined after collecting and analyzing a full year of post-onset EHR data.

To establish a rigorous labeling methodology, we define several key concepts:

- PD onset datetime: The timestamp of the patient's PD onset HbA1c record, serving as the prediction time when the system collects all available historical EHR data.
- Future EHRs: All health records from the PD onset datetime to the end of the prediction horizon (12 months for a one-year prediction period).
- Clinical Events: We extract diabetes events (HbA1c ≥6.5%, ICD-10 diagnosis codes, anti-diabetic medications) and non-diabetes events (primarily HbA1c < 6.5%) from Future EHRs.

In contrast to prior foundational studies that examined diabetes events alone, our approach adopts a more broader perspective by considering both diabetes and non-diabetes events, along with their timing in the patient's record. This integrated analysis enables a more informed inference of the underlying ground truth status of PD onset patients and enables appropriate labeling.

Enhanced Approaches for *diabetic* Patient Identification and Noise Detection

Some earlier studies defined diabetes progression using narrow criteria. For example, Aoki et al.[4] defined *diabetic* labels based on an HbA1c threshold of ≥6.5%, while Han et al.[3] and Liu et al.[2] restricted their criteria to glucose values alone. While these definitions offer simplicity and consistency, they may miss important clinical nuances. For example, patients might be prescribed diabetes medications before their HbA1c reaches the diagnostic threshold, particularly with other risk factors or symptoms. Second, some patients may maintain HbA1c levels below 6.5% precisely because they are already receiving medication management. Third, medication prescriptions often represent a physician's clinical judgment based on comprehensive patient assessment beyond laboratory values.

We enhance existing labeling frameworks by also identifying early intervention cases—patients initially classified as PD onset who receive diabetes-related treatment within one month. These atypical early interventions might indicate that the patient was diabetics but misclassified as PD

onset due to limited EHR data. Consequently, we exclude these misclassified cases from our final dataset to maintain analytical integrity—an adjustment that contribute to existing literature.

Enhanced Approaches for *non-diabetic* Patient Identification

Classifying low-risk patients in real-world EHR data presents unique challenges. For example, Zueger et al.[6] treated patients without follow-up records as *non-diabetic* cases, a strategy that may unintentionally conflate different clinical scenarios. We acknowledge that missing follow-up data could indicate various scenarios—from successful disease management to complete loss of care—and shouldn't automatically be interpreted as *non-diabetic*. Therefore, our methodology labels patients without any recorded events as "missing labels," mitigating this source of error.

Traditional methods typically classify patients as *non-diabetic* if their Future EHRs contain non-diabetes events and lack diabetes events. However, this approach may not always consider the temporal relevance to those events. For example, a normal HbA1c reading at the 3rd month or the 18th month provides limited evidence about the patient's disease at the end of the evaluation period (one year after PD onset, i.e., months 10-14).

We address this by emphasizing non-diabetes events that occur between months 10-14 (near the end of our one-year prediction horizon). This time-aware strategy improves label precision by prioritizing clinical evidence that are most indicative of the target time point.

1.4 Patient Subgroup Analysis

Previous PD2D studies have provided valuable tools for predicting diabetes risk, however, opportunities remain to further explore the clinical profiles and their progression patterns within different risk-based subgroups. Understanding these trajectories is essential for clinicians seeking to personalize prediabetes management rather than relying on a one-size-fits-all approach.

In a related study, Ahlqvist et al.[7] conducted a comprehensive analysis of diabetes-onset subgroups, demonstrating how distinct patient profiles affect disease progression. Extending this concept to prediabetes may uncover important differences among patient groups, potentially enabling more precise interventions. By examining risk subgroups based on clinical profiles, clinicians could identify subgroups characterized by unique patterns and progression rates, allowing for targeted preventive strategies tailored to individual needs.

Beyond improving patient care, this analysis could provide critical insights into the mechanisms driving diabetes progression. Identifying how specific clinical factors contribute to risk would refine our understanding of disease development, ultimately enhancing early intervention efforts and optimizing resource allocation. Personalized risk assessments could lead to better patient communication, increased engagement, and greater adherence to preventive measures, improving long-term health outcomes.

Tracking the progression to diabetes within these subgroups could also serve as a valuable real-world validation of the methodology. If observed outcomes align with subgroup-based predictions,

this would reinforce the practical utility of this approach in clinical care. This iterative feedback loop—where real-world outcomes refine subgroup definitions—could further enhance their clinical utility.

In this study, we aim to support this line of inquiry by analyzing clinical profiles and their progression to diabetes outcomes across different risk subgroups. By examining how patients transition over time, we aim to validate the distinctiveness and clinical relevance of these subgroups, providing a stronger foundation for integrating risk-based strategies into prediabetes management.

2. Diabetes and None-Diabetes Events

2.1 Definitions of Diabetes and Non-diabetes Events.

We extract diabetes and non-diabetes events from Future EHRs to establish evidence of disease progression. Diabetes events are defined as meeting any of the following criteria: laboratory evidence of HbA1c ≥6.5%, which is the standard diagnostic threshold recommended by the American Diabetes Association (ADA); formal diabetes diagnosis codes, including ICD-10 codes E11.* (Type 2 diabetes mellitus) and related subcategories; or the prescription of diabetes-specific medications, including but not limited to metformin, sulfonylureas, DPP-4 inhibitors, GLP-1 receptor agonists, SGLT2 inhibitors, thiazolidinediones, or insulin therapy. Among these three diabetes indicators, HbA1c measurements provide the most objective evidence, whereas diagnosis codes and medication prescriptions incorporate both clinical judgment and patient preferences. These latter indicators may not fully reflect the underlying physiological state of diabetes. However, in this study, we assign equal weight to all three indicators in our analysis.

Conversely, non-diabetes events are primarily characterized by HbA1c measurements in the prediabetic range (5.7% to 6.4%) or normal range (<5.7%). However, the presence of non-diabetes events alone does not necessarily confirm a non-diabetic status, as patients may use medications or other treatment methods to manage HbA1c levels below 6.5%. Therefore, both the absence of diabetes events and the presence of non-diabetes events must be considered to accurately assess disease status.

2.2 Diabetes Medication List

Our medication selection process from the EHR system is designed to identify diabetes medications based on their inclusion in either the Medication Category (medication categories from Cerner Multum) or the Medication Order Mnemonic. Any medication that appears in either of these lists is classified as a diabetes medication. The Medication Category defines broad pharmacological or therapeutic classes, while the Medication Order Mnemonic contains specific medication order mnemonics.

Medication Category	Mnemonic
Sulfonylureas	glipiZIDE, glyBURIDE, glimepiride, chlorproPAMIDE, glipiZIDE-metFORMIN, glyBURIDE-metFORMIN

Insulin	insulin lispro, insulin glargine, insulin regular, insulin aspart, insulin detemir, insulin degludec, insulin glulisine, insulin aspart-insulin aspart protamine, insulin isophane, insulin isophane-insulin regular, insulin regular 100 units, insulin lispro-insulin lispro protamine, insulin glargine-lixisenatide, insulin regular + Sodium Chloride 0.9%, insulin regular 3 units, insulin regular 1 units, insulin regular 1000 units +, insulin regular (ANES), insulin regular + sodium chloride premixes, insulin regular 300 units + sodium chloride premixes
Biguanides	metFORMIN, metFORMIN-sitaGLIPtin, metFORMIN-repaglinide, linagliptin-metFORMIN, metFORMIN-pioglitazone, metFORMIN-rosiglitazone, metFORMIN-saxagliptin, empagliflozin/linagliptin/metformin, dapagliflozin-metFORMIN, canagliflozin-metFORMIN, ertugliflozin-metFORMIN
GLP-1 Receptor Agonists	dulaglutide, semaglutide, liraglutide, tirzepatide, exenatide, albiglutide, insulin degludec-liraglutide
Dipeptidyl Peptidase 4 Inhibitors	SITagliptin, saxagliptin, alogliptin, linagliptin, alogliptin-metFORMIN
SGLT-2 Inhibitors	empagliflozin, dapagliflozin, canagliflozin, ertugliflozin, bexagliflozin, empagliflozin-linagliptin
Antidiabetic Combinations	metFORMIN-sitaGLIPtin, linagliptin-metFORMIN, empagliflozin-metFORMIN, dapagliflozin-metFORMIN, empagliflozin-linagliptin, glipiZIDE-metFORMIN, glyBURIDE-metFORMIN, metFORMIN-pioglitazone, metFORMIN-repaglinide, metFORMIN-rosiglitazone, empagliflozin/linagliptin/metformin
Thiazolidinediones	pioglitazone, rosiglitazone, glimepiride-rosiglitazone
Alpha-Glucosidase Inhibitors	acarbose

Meglitinides	repaglinide, nateglinide
Amylin Analogs	pramlintide

2.3 Diagnosis List

According to the U.S. Department of Health and Human Services (HHS), October 1, 2015, marked the compliance deadline for healthcare providers, health plans, and healthcare clearinghouses to transition to the ICD-10-CM/PCS coding system. While our data primarily reflects the year starting from 2017, we continue to include ICD-9-CM codes as a supplementary reference alongside ICD-10-CM codes.

Diagnosis	ICD-9-CM	ICD-10-CM Parent Code
Prediabetes	790.29	R73.03
Type 1 diabetes mellitus	· 250.01<br>· 250.03<br>· 250.11<br>· 250.13<br>· 250.21<br>· 250.23<br>· 250.31<br>· 250.33<br>· 250.41<br>· 250.43<br>· 250.51<br>· 250.53<br>· 250.61<br>· 250.63<br>· 250.71<br>· 250.73<br>· 250.81<br>· 250.83<br>· 250.91<br>· 250.93	E10
Type 2 diabetes mellitus	· 250.00<br>· 250.02<br>· 250.10<br>· 250.12<br>· 250.20	E11

	· 250.22<br>· 250.30<br>· 250.32<br>· 250.40<br>· 250.42<br>· 250.50<br>· 250.52<br>· 250.60<br>· 250.62<br>· 250.70<br>· 250.72<br>· 250.80<br>· 250.82<br>· 250.90<br>· 250.92	

3. Input EHR Features

The input features are derived from patients' EHRs prior to PD onset. The observation period for this study is defined as a one-year look-back from the date of the PD onset. The EHR data encompass laboratory and vital sign measurements, including BMI, ALT, A1C, LDL, HDL, systolic blood pressure (SBP), and diastolic blood pressure (DBP); lifestyle factors such as smoking status, alcohol consumption, and physical activity; medication history and diagnostic codes; clinical note types; and patient demographic information. All EHR data observed during this period are incorporated into the analysis. The EHR features are represented by the aggregated mean values of these records over the observation period to ensure a comprehensive and standardized representation of patient health status prior to PD onset.

The codes for developing the features are listed as below:

```
CF_to_CFBf1MEMRInfo = {
    'cf.Bf1M_LabVital_Nume':{
        'CO_to_COName': {
            'co.Bf1M_A1C_Nume':  'phi.AggMeanFeat-hP.rA1C.fNume.cBf1M',
            'co.Bf1M_BMI_Nume':  'phi.AggMeanFeat-hP.rCEBMI.fNume.cBf1M',
            'co.Bf1M_ALT_Nume':  'phi.AggMeanFeat-hP.rCEALT.fNume.cBf1M',
            'co.Bf1M_HDL_Nume':  'phi.AggMeanFeat-hP.rCEHDL.fNume.cBf1M',
            'co.Bf1M_LDL_Nume':  'phi.AggMeanFeat-hP.rCELDL.fNume.cBf1M',
            'co.Bf1M_DBP_Nume':  'phi.AggMeanFeat-hP.rCEDBP.fNume.cBf1M',
            'co.Bf1M_SBP_Nume':  'phi.AggMeanFeat-hP.rCESBP.fNume.cBf1M',
        },
        'Gamma': 'CatTknSet',
    },
    'cf.Bf1M_LifeStyle_Cate': {
        'CO_to_COName': {
            'co.Bf1M_Alcohol_Cate':    'phi.AggMeanFeat-hP.rCEAlcohol.fCate.cBf1M',
            'co.Bf1M_Exercise_Cate':   'phi.AggMeanFeat-hP.rCEExercise.fCate.cBf1M',
            'co.Bf1M_Diet_Cate':       'phi.AggMeanFeat-hP.rCEDiet.fCate.cBf1M',
            'co.Bf1M_Smoking_Cate':    'phi.AggMeanFeat-hP.rCESmoking.fCate.cBf1M',
        },
        'Gamma': 'CatTknSet',
    },
    'cf.Bf1M_MedDiag_Cate': {
        'CO_to_COName': {
            'co.Bf1M_OrderMed_Cate':    'phi.AggMeanFeat-hP.rOrderMed.fCate.cBf1M',
            'co.Bf1M_Diag_Cate':        'phi.AggMeanFeat-hP.rDiag.fCate.cBf1M',
            'co.Bf1M_Diag_Prefix3Cate':  'phi.AggMeanFeat-hP.rDiag.fPrefix3Cate.cBf1M',
            # NoteType.
```

```
        },
        'Gamma': 'CatTknSet',
    },
    'cf.Bf1M_LabVital_RecNum':{
        'CO_to_COName': {
            'co.Bf1M_A1C_RN':  'phi.RecNum-hP.rA1C.cBf1M',
            'co.Bf1M_BMI_RN':  'phi.RecNum-hP.rCEBMI.cBf1M',
            'co.Bf1M_ALT_RN':  'phi.RecNum-hP.rCEALT.cBf1M',
            'co.Bf1M_HDL_RN':  'phi.RecNum-hP.rCEHDL.cBf1M',
            'co.Bf1M_LDL_RN':  'phi.RecNum-hP.rCELDL.cBf1M',
            'co.Bf1M_DBP_RN':  'phi.RecNum-hP.rCEDBP.cBf1M',
            'co.Bf1M_SBP_RN':  'phi.RecNum-hP.rCESBP.cBf1M',
        },
        'Gamma': 'CatTknSet',
    },
    'cf.Bf1M_LifeStyle_RecNum': {
        'CO_to_COName': {
            'co.Bf1M_Alcohol_RN':   'phi.RecNum-hP.rCEAlcohol.cBf1M',
            'co.Bf1M_Exercise_RN':  'phi.RecNum-hP.rCEExercise.cBf1M',
            'co.Bf1M_Diet_RN':      'phi.RecNum-hP.rCEDiet.cBf1M',
            'co.Bf1M_Smoking_RN':   'phi.RecNum-hP.rCESmoking.cBf1M',
        },
        'Gamma': 'CatTknSet',
    },
    'cf.Bf1M_MedDiag_RecNum':{
        'CO_to_COName': {
            'co.Bf1M_Diag_RN':      'phi.RecNum-hP.rDiag.cBf1M',
            'co.Bf1M_Med_RN':       'phi.RecNum-hP.rOrderMed.cBf1M',
        },
         'Gamma': 'CatTknSet',
    }
}
```

The feature engineering process for EHRs demonstrates a sophisticated approach to capturing patient health status through multiple dimensions. This system creates a comprehensive patient profile by examining data from the past month (indicated by "Bf1M" - Before 1 Month), organizing health information into three primary categories: laboratory/vital measurements, lifestyle factors, and medical/diagnostic information.

The laboratory and vital signs features form the quantitative foundation of the patient profile. These include critical health indicators such as HbA1c (a measure of blood glucose control over

time), BMI (Body Mass Index for weight status), ALT (liver function), HDL and LDL cholesterol levels (cardiovascular health indicators), and blood pressure measurements (both systolic and diastolic). For each of these measurements, the system calculates mean values over the one-month period, providing a stable representation of the patient's physiological status. Additionally, it tracks the frequency of these measurements, which can indicate the intensity of patient monitoring and healthcare engagement.

Lifestyle factors represent behavioral determinants of health outcomes. The system categorizes patient behaviors related to alcohol consumption, exercise habits, dietary patterns, and smoking status. These lifestyle features are particularly valuable as they capture modifiable risk factors that significantly impact health outcomes. The frequency of lifestyle-related records may also indicate the level of attention being paid to behavioral health interventions and patient engagement with lifestyle modification programs.

The medical and diagnostic features encompass the clinical intervention and disease status aspects of patient care. This category includes ordered medications, which reflect the therapeutic approach, and diagnostic codes at both detailed and broader levels (using full codes and three-character prefixes). The inclusion of both full diagnostic codes and their prefixes allows for analysis at different levels of clinical specificity. The system tracks not only the presence of diagnoses and medications but also their frequency, providing insight into the complexity and intensity of medical care.

An important aspect of this feature engineering approach is its dual focus on values and frequencies. For each category, the system generates features based on actual measurements or classifications (through "Nume" for numeric values and "Cate" for categorical data) and the number of records ("RecNum"). This dual approach provides a more complete picture of patient health status by capturing both the clinical values and the patterns of healthcare utilization and monitoring.

The use of aggregated mean features ("AggMeanFeat") for numerical values helps smooth out potential noise in the measurements while maintaining the central tendency of the health indicators. The categorical token set approach ("CatTknSet") suggests a standardized method for handling non-numeric data, ensuring consistent representation of qualitative health information across the feature set.

This comprehensive feature engineering strategy creates a rich, multidimensional representation of patient health status, combining objective clinical measurements, behavioral factors, and healthcare utilization patterns. The resulting feature set supports sophisticated health analytics applications, potentially enabling better prediction of health outcomes, risk stratification, and personalized care recommendations.

4. Model Building and Evaluation

We developed and evaluated our PD2D risk prediction model using the machine learning approaches of XGBoost as our primary algorithm. XGBoost (eXtreme Gradient Boosting) is an efficient implementation of gradient-boosted decision trees that sequentially corrects prediction errors through gradient descent optimization[8]. This algorithm was chosen for its proven performance in handling high-dimensional data and its ability to capture complex non-linear relationships. The implementation was carried out using the scikit-learn framework, which provides robust tools for machine learning model development and evaluation. EHR data was preprocessed into high-dimensional sparse feature vectors, serving as input to our models. This transformation preserved the rich information content while maintaining computational efficiency.

To optimize the model's predictive performance, we performed hyperparameter tuning with the grid search method. The tuning process involved systematically evaluating different configurations of key hyperparameters, including the number of boosting rounds (n_estimators), learning rate (eta), tree depth (max_depth), and early stopping criteria. Bayesian optimization was employed to efficiently explore the search space by iteratively refining hyperparameter choices based on past evaluations. The performance of each configuration was assessed using cross-validation, with the log-loss metric serving as the primary evaluation criterion. The final hyperparameter settings, which yielded the best trade-off between predictive accuracy and generalizability, were determined as follows: max_depth = 10, random_state = 42, n_estimators = 1000, learning_rate = 0.01, objective = 'binary:logistic', early_stopping_rounds = 10, and eval_metric = 'logloss'. These optimized hyperparameters were used to train the final model for PD2D risk prediction.

We employed multiple complementary metrics to comprehensively evaluate model performance.

AUC. The AUC serves as our primary evaluation metric, providing a threshold-independent assessment of the model's discriminative ability. We calculate empirical 95% confidence intervals using stratified bootstrap with 2,000 replications to ensure robust uncertainty estimation. The AUC ranges from 0 to 1, with 1 representing perfect discrimination and 0.5 indicating random chance performance.

Accuracy with Threshold. We evaluate the model's overall classification accuracy using predefined clinically relevant thresholds. For HbA1c predictions, we use a threshold of 6.0%, while for the AI risk score, we employ a threshold of 0.5. The accuracy is calculated as the proportion of correct predictions (both true positives and true negatives) among all predictions, providing a balanced measure of the model's performance across all classes.

Precision at Top 10%. This metric evaluates the model's ability to identify high-risk individuals by focusing on the subset of patients with the highest predicted risk scores. We rank all patients by their predicted risk scores and calculate the proportion of true positives among the top 10% of predictions. This metric is particularly relevant for clinical applications where identifying the highest-risk patients is crucial for targeted interventions.

Precision at Bottom 10%. Similar to the top 10% precision, this metric assesses the model's performance on the lowest-risk subset. We rank patients by their predicted risk scores and calculate the proportion of true positives among the bottom 10% of predictions. This helps evaluate the model's ability to correctly identify low-risk individuals, which is valuable for resource allocation and intervention planning.

5. Patient Subgroup Description Table

Table S1. Patient characteristics for different risk level patients on training set (2018-2020 cohort) with the format: Median (Q1-Q3) or percentage.

	**Low-Risk**	**Medium-Risk**	**High-Risk**
N	3758	2705	1165
Prediabetes onset HbA1c (%)	5.80 (5.70-5.90)	6.00 (5.80-6.10)	6.20 (6.00-6.30)
Age (year)	55.94 (46.80-63.89)	58.69 (48.17-68.97)	62.88 (54.00-72.50)
BMI (kg/m^2)	30.48 (26.69-35.30)	32.91 (27.88-39.60)	31.56 (26.78-37.83)
ALT (U/L)	19.00 (14.00-27.00)	21.00 (16.00-31.00)	24.00 (18.00-35.17)
HDL (mg/dL)	53.00 (44.00-64.00)	47.00 (39.00-58.00)	45.00 (36.00-57.00)
LDL (mg/dL)	112.00 (91.00-134.00)	105.00 (78.00-132.00)	89.00 (65.00-120.00)
DBP (mmHg)	80.00 (72.00-85.00)	80.00 (73.30-87.67)	79.28 (71.11-90.00)
SBP (mmHg)	127.00 (118.00-138.00)	132.00 (121.00-146.00)	136.38 (122.72-154.41)
Black	51.65%	50.94%	55.97%
Other	13.04%	9.94%	9.87%
White	35.31%	39.11%	34.16%
Female	62.13%	57.60%	51.42%
Male	37.87%	42.40%	48.58%

Table S2. Patient characteristics for different risk level patients on the evaluation set (2021-2022 cohort) with the format: Median (Q1-Q3) or percentage.

	**Low-Risk**	**Medium-Risk**	**High-Risk**
N	3656	2439	713
Prediabetes onset HbA1c (%)	5.80 (5.70-5.90)	6.00 (5.80-6.10)	6.20 (5.90-6.30)
Age (year)	54.92 (44.39-64.44)	57.56 (44.94-68.31)	64.38 (51.39-74.50)
BMI (kg/m^2)	29.56 (25.97-34.20)	34.29 (29.08-40.92)	30.89 (26.04-38.12)
ALT (U/L)	19.00 (14.00-26.00)	20.00 (14.62-30.00)	23.00 (16.00-37.00)
HDL (mg/dL)	54.00 (46.00-66.00)	47.00 (40.00-58.00)	41.00 (33.00-49.75)
LDL (mg/dL)	119.00 (97.00-143.00)	113.00 (85.00-142.00)	87.00 (65.00-110.00)
DBP (mmHg)	80.00 (73.50-84.50)	81.50 (74.00-88.00)	79.62 (71.43-88.25)
SBP (mmHg)	126.00 (118.00-136.00)	132.00 (121.19-146.00)	138.00 (124.50-154.00)
Black	37.42%	46.66%	52.88%
Other	19.56%	14.51%	12.62%
White	43.03%	38.83%	34.50%
Female	62.83%	63.26%	51.47%
Male	37.17%	36.74%	48.53%

6. KM Survival Table for PD onset HbA1C subgroups

6.1 2018-2020 Cohort across Prediabetes onset HbA1c.

Table S3: The 2018-2020 Cohort with Prediabetes onset HbA1c of 5.7-5.9.

	removed	observed	censored	entrance	at_risk	5.7-5.9	lower_0.95	upper_0.95
0	0	0	0	4766	4766	1	1	1
1	63	63	0	0	4766	0.9868	0.9831	0.9897
2	124	124	0	0	4703	0.9608	0.9549	0.9659
3	94	94	0	0	4579	0.9410	0.9340	0.9474
4	83	83	0	0	4485	0.9236	0.9157	0.9308
5	83	83	0	0	4402	0.9062	0.8976	0.9141
6	70	70	0	0	4319	0.8915	0.8824	0.9000
7	71	71	0	0	4249	0.8766	0.8670	0.8856
8	72	72	0	0	4178	0.8615	0.8514	0.8710
9	55	55	0	0	4106	0.8500	0.8395	0.8598
10	48	48	0	0	4051	0.8399	0.8292	0.8500
11	160	68	92	0	4003	0.8256	0.8146	0.8361
12	184	50	134	0	3843	0.8149	0.8036	0.8256
13	314	30	284	0	3659	0.8082	0.7967	0.8191
14	225	21	204	0	3345	0.8031	0.7915	0.8142
15	56	13	43	0	3120	0.7998	0.7881	0.8110
16	19	9	10	0	3064	0.7974	0.7857	0.8087
17	30	13	17	0	3045	0.7940	0.7822	0.8054
18	27	11	16	0	3015	0.7911	0.7792	0.8026
19	39	13	26	0	2988	0.7877	0.7756	0.7992
20	30	13	17	0	2949	0.7842	0.7721	0.7958
21	18	9	9	0	2919	0.7818	0.7696	0.7935
22	34	10	24	0	2901	0.7791	0.7668	0.7909
23	19	7	12	0	2867	0.7772	0.7649	0.7890

Table S4. The 2018-2020 Cohort with Prediabetes onset HbA1c of 6.0-6.4

	removed	observed	censored	entrance	at_risk	6.0-6.4	lower_0.95	upper_0.95
0	0	0	0	2862	2862	1	1	1
1	129	129	0	0	2862	0.9549	0.9467	0.9619
2	164	164	0	0	2733	0.8976	0.8859	0.9082
3	121	121	0	0	2569	0.8553	0.8419	0.8677
4	164	164	0	0	2448	0.7980	0.7829	0.8123
5	116	116	0	0	2284	0.7575	0.7414	0.7728
6	111	111	0	0	2168	0.7187	0.7019	0.7348
7	122	122	0	0	2057	0.6761	0.6586	0.6929
8	100	100	0	0	1935	0.6412	0.6233	0.6584
9	92	92	0	0	1835	0.6090	0.5909	0.6266
10	90	90	0	0	1743	0.5776	0.5592	0.5954
11	121	85	36	0	1653	0.5479	0.5294	0.5659
12	129	76	53	0	1532	0.5207	0.5022	0.5388
13	155	49	106	0	1403	0.5025	0.4840	0.5207
14	79	23	56	0	1248	0.4932	0.4747	0.5115
15	30	14	16	0	1169	0.4873	0.4687	0.5057
16	12	10	2	0	1139	0.4831	0.4644	0.5014
17	21	17	4	0	1127	0.4758	0.4571	0.4942
18	25	13	12	0	1106	0.4702	0.4515	0.4886
19	19	10	9	0	1081	0.4658	0.4471	0.4843
20	17	9	8	0	1062	0.4619	0.4432	0.4804
21	20	17	3	0	1045	0.4544	0.4356	0.4729
22	15	6	9	0	1025	0.4517	0.4330	0.4703
23	18	13	5	0	1010	0.4459	0.4271	0.4645

6.2 2021-2022 Cohort across Prediabetes onset HbA1c.

Table S5: The 2021-2022 Cohort with Prediabetes onset HbA1c of 5.7-5.9.

	removed	observed	censored	entrance	at_risk	5.7-5.9	lower_0.95	upper_0.95
0	0	0	0	4683	4683	1	1	1
1	36	36	0	0	4683	0.9923	0.9894	0.9944
2	113	113	0	0	4647	0.9682	0.9627	0.9728
3	83	83	0	0	4534	0.9505	0.9438	0.9563
4	88	88	0	0	4451	0.9317	0.9241	0.9385
5	72	72	0	0	4363	0.9163	0.9080	0.9239
6	72	72	0	0	4291	0.9009	0.8920	0.9091
7	80	80	0	0	4219	0.8838	0.8743	0.8927
8	74	74	0	0	4139	0.8680	0.8580	0.8774
9	70	70	0	0	4065	0.8531	0.8426	0.8629
10	50	50	0	0	3995	0.8424	0.8317	0.8525
11	261	53	208	0	3945	0.8311	0.8201	0.8415
12	447	58	389	0	3684	0.8180	0.8066	0.8288
13	1075	47	1028	0	3237	0.8061	0.7944	0.8173
14	601	36	565	0	2162	0.7927	0.7804	0.8045
15	131	19	112	0	1561	0.7831	0.7701	0.7954
16	46	13	33	0	1430	0.7759	0.7625	0.7887
17	63	18	45	0	1384	0.7658	0.7518	0.7793
18	77	20	57	0	1321	0.7543	0.7394	0.7684
19	95	15	80	0	1244	0.7452	0.7298	0.7598
20	95	19	76	0	1149	0.7328	0.7167	0.7482
21	84	14	70	0	1054	0.7231	0.7064	0.7391
22	64	12	52	0	970	0.7142	0.6969	0.7307
23	52	5	47	0	906	0.7102	0.6927	0.7270
24	85	11	74	0	854	0.7011	0.6829	0.7184

Table S6: The 2021-2022 Cohort with Prediabetes onset HbA1c of 6.0-6.4

	removed	observed	censored	entrance	at_risk	6.0-6.4	lower_0.95	upper_0.95
0	0	0	0	2125	2125	1	1	1
1	91	91	0	0	2125	0.9572	0.9477	0.9650
2	128	128	0	0	2034	0.8969	0.8832	0.9091
3	90	90	0	0	1906	0.8546	0.8389	0.8689
4	105	105	0	0	1816	0.8052	0.7877	0.8214
5	81	81	0	0	1711	0.7671	0.7485	0.7845
6	69	69	0	0	1630	0.7346	0.7153	0.7528
7	81	81	0	0	1561	0.6965	0.6764	0.7155
8	66	66	0	0	1480	0.6654	0.6449	0.6850
9	69	69	0	0	1414	0.6329	0.6121	0.6530
10	61	61	0	0	1345	0.6042	0.5831	0.6247
11	143	57	86	0	1284	0.5774	0.5561	0.5981
12	177	58	119	0	1141	0.5481	0.5265	0.5690
13	279	31	248	0	964	0.5304	0.5087	0.5516
14	181	24	157	0	685	0.5119	0.4897	0.5336
15	26	11	15	0	504	0.5007	0.4780	0.5229
16	12	5	7	0	478	0.4954	0.4726	0.5179
17	20	9	11	0	466	0.4859	0.4626	0.5087
18	45	17	28	0	446	0.4674	0.4434	0.4910
19	29	7	22	0	401	0.4592	0.4349	0.4832
20	38	9	29	0	372	0.4481	0.4233	0.4725
21	33	12	21	0	334	0.4320	0.4065	0.4572
22	34	11	23	0	301	0.4162	0.3900	0.4422
23	23	5	18	0	267	0.4084	0.3818	0.4348
24	19	5	14	0	244	0.4000	0.3730	0.4269

7. KM Survival Table for PD onset HbA1C subgroups

7.1 2018-2020 Cohort across Risk Subgroups.

Table S7. 2018-2020 Cohort, prediabetes-patient classified as the high-risk subgroup.

	removed	observed	censored	entrance	at_risk	High-Risk	lower_0.95	upper_0.95
0	0	0	0	1197	1197	1	1	1
1	138	138	0	0	1197	0.8847	0.8652	0.9015
2	160	160	0	0	1059	0.7510	0.7255	0.7746
3	106	106	0	0	899	0.6625	0.6349	0.6885
4	127	127	0	0	793	0.5564	0.5278	0.5840
5	96	96	0	0	666	0.4762	0.4476	0.5042
6	89	89	0	0	570	0.4018	0.3740	0.4295
7	87	87	0	0	481	0.3292	0.3027	0.3559
8	85	85	0	0	394	0.2581	0.2337	0.2832
9	68	68	0	0	309	0.2013	0.1791	0.2245
10	65	65	0	0	241	0.1470	0.1276	0.1677
11	59	57	2	0	176	0.0994	0.0833	0.1172
12	52	52	0	0	117	0.0552	0.0432	0.0692
13	20	17	3	0	65	0.0408	0.0306	0.0531
14	3	1	2	0	45	0.0399	0.0298	0.0521
15	4	4	0	0	42	0.0361	0.0265	0.0479
16	1	1	0	0	38	0.0351	0.0256	0.0468
17	1	1	0	0	37	0.0342	0.0248	0.0458
18	2	1	1	0	36	0.0332	0.0240	0.0447
19	2	1	1	0	34	0.0323	0.0232	0.0436
20	1	0	1	0	32	0.0323	0.0232	0.0436
22	1	1	0	0	31	0.0312	0.0223	0.0425
23	1	1	0	0	30	0.0302	0.0214	0.0413
24	4	1	3	0	29	0.0291	0.0205	0.0402

Table S8. 2018-2020 Cohort, prediabetes-patient classified as the low-risk subgroup.

	removed	observed	censored	entrance	at_risk	Low-Risk	lower_0.95	upper_0.95
0	0	0	0	3804	3804	1	1	1
1	4	4	0	0	3804	0.9989	0.9972	0.9996
2	8	8	0	0	3800	0.9968	0.9945	0.9982
3	3	3	0	0	3792	0.9961	0.9935	0.9976
4	5	5	0	0	3789	0.9947	0.9919	0.9966
5	5	5	0	0	3784	0.9934	0.9903	0.9956
6	3	3	0	0	3779	0.9926	0.9894	0.9949
7	6	6	0	0	3776	0.9911	0.9875	0.9936
8	3	3	0	0	3770	0.9903	0.9866	0.9929
9	3	3	0	0	3767	0.9895	0.9857	0.9923
10	4	4	0	0	3764	0.9884	0.9845	0.9914
11	68	7	61	0	3760	0.9866	0.9824	0.9898
12	107	4	103	0	3692	0.9855	0.9812	0.9889
13	303	26	277	0	3585	0.9784	0.9732	0.9826
14	190	17	173	0	3282	0.9733	0.9676	0.9781
15	48	9	39	0	3092	0.9705	0.9644	0.9755
16	14	7	7	0	3044	0.9682	0.9619	0.9735
17	31	15	16	0	3030	0.9635	0.9567	0.9692
18	32	12	20	0	2999	0.9596	0.9525	0.9657
19	40	14	26	0	2967	0.9551	0.9476	0.9615
20	26	11	15	0	2927	0.9515	0.9437	0.9582
21	20	11	9	0	2901	0.9479	0.9398	0.9549
22	35	10	25	0	2881	0.9446	0.9363	0.9518
23	19	8	11	0	2846	0.9419	0.9334	0.9494
24	31	10	21	0	2827	0.9386	0.9299	0.9463

Table S9. 2018-2020 Cohort, prediabetes-patient classified as the Medium-risk subgroup.

	removed	observed	censored	entrance	at_risk	Medium-Risk	lower_0.95	upper_0.95
0	0	0	0	2627	2627	1	1	1
1	50	50	0	0	2627	0.9810	0.9750	0.9855
2	120	120	0	0	2577	0.9353	0.9252	0.9441
3	106	106	0	0	2457	0.8949	0.8826	0.9061
4	115	115	0	0	2351	0.8512	0.8370	0.8642
5	98	98	0	0	2236	0.8139	0.7984	0.8282
6	89	89	0	0	2138	0.7800	0.7636	0.7953
7	100	100	0	0	2049	0.7419	0.7247	0.7582
8	84	84	0	0	1949	0.7099	0.6922	0.7269
9	76	76	0	0	1865	0.6810	0.6628	0.6985
10	69	69	0	0	1789	0.6547	0.6362	0.6726
11	154	89	65	0	1720	0.6209	0.6020	0.6391
12	154	70	84	0	1566	0.5931	0.5740	0.6117
13	146	36	110	0	1412	0.5780	0.5587	0.5967
14	111	26	85	0	1266	0.5661	0.5467	0.5850
15	34	14	20	0	1155	0.5593	0.5398	0.5782
16	16	11	5	0	1121	0.5538	0.5342	0.5728
17	19	14	5	0	1105	0.5468	0.5271	0.5659
18	18	11	7	0	1086	0.5412	0.5215	0.5605
19	16	8	8	0	1068	0.5372	0.5174	0.5565
20	20	11	9	0	1052	0.5315	0.5117	0.5510
21	18	15	3	0	1032	0.5238	0.5039	0.5433
22	13	5	8	0	1014	0.5212	0.5013	0.5408
23	18	12	6	0	1001	0.5150	0.4950	0.5346
24	14	4	10	0	983	0.5129	0.4928	0.5326

7.2 2021-2022 Cohort across Risk Subgroups.

Table S10. 2021-2022 Cohort, prediabetes-patient classified as the high-risk subgroup.

	removed	observed	censored	entrance	at_risk	High-Risk	lower_0.95	upper_0.95
0	0	0	0	719	719	1	1	1
1	72	72	0	0	719	0.8999	0.8755	0.9197
2	83	83	0	0	647	0.7844	0.7525	0.8127
3	62	62	0	0	564	0.6982	0.6632	0.7303
4	61	61	0	0	502	0.6134	0.5767	0.6479
5	55	55	0	0	441	0.5369	0.4997	0.5725
6	32	32	0	0	386	0.4924	0.4553	0.5283
7	36	36	0	0	354	0.4423	0.4057	0.4782
8	34	34	0	0	318	0.3950	0.3592	0.4306
9	30	30	0	0	284	0.3533	0.3185	0.3882
10	26	26	0	0	254	0.3171	0.2834	0.3513
11	36	21	15	0	228	0.2879	0.2552	0.3213
12	58	31	27	0	192	0.2414	0.2105	0.2735
13	39	11	28	0	134	0.2216	0.1913	0.2533
14	36	10	26	0	95	0.1983	0.1683	0.2301
15	8	3	5	0	59	0.1882	0.1579	0.2206
16	1	1	0	0	51	0.1845	0.1540	0.2172
17	2	1	1	0	50	0.1808	0.1502	0.2137
18	6	5	1	0	48	0.1620	0.1310	0.1959
19	1	0	1	0	42	0.1620	0.1310	0.1959
20	1	0	1	0	41	0.1620	0.1310	0.1959
21	2	0	2	0	40	0.1620	0.1310	0.1959
22	6	4	2	0	38	0.1449	0.1137	0.1798
23	3	1	2	0	32	0.1404	0.1092	0.1755
24	3	1	2	0	29	0.1356	0.1043	0.1709

Table S11. 2021-2022 Cohort, prediabetes-patient classified as the low-risk subgroup.

	removed	observed	censored	entrance	at_risk	Low Risk	lower_0.95	upper_0.95
0	0	0	0	3677	3677	1	1	1
1	2	2	0	0	3677	0.9995	0.9978	0.9999
2	35	35	0	0	3675	0.9899	0.9861	0.9927
3	33	33	0	0	3640	0.9810	0.9760	0.9849
4	36	36	0	0	3607	0.9712	0.9652	0.9761
5	32	32	0	0	3571	0.9625	0.9558	0.9681
6	25	25	0	0	3539	0.9557	0.9485	0.9619
7	33	33	0	0	3514	0.9467	0.9389	0.9535
8	36	36	0	0	3481	0.9369	0.9286	0.9443
9	32	32	0	0	3445	0.9282	0.9194	0.9361
10	26	26	0	0	3413	0.9211	0.9119	0.9294
11	197	32	165	0	3387	0.9124	0.9028	0.9211
12	319	21	298	0	3190	0.9064	0.8965	0.9154
13	975	37	938	0	2871	0.8947	0.8842	0.9043
14	532	27	505	0	1896	0.8820	0.8705	0.8925
15	109	16	93	0	1364	0.8717	0.8592	0.8831
16	33	10	23	0	1255	0.8647	0.8515	0.8768
17	50	15	35	0	1222	0.8541	0.8399	0.8671
18	69	14	55	0	1172	0.8439	0.8289	0.8577
19	89	11	78	0	1103	0.8355	0.8197	0.8500
20	82	15	67	0	1014	0.8231	0.8063	0.8386
21	77	12	65	0	932	0.8125	0.7948	0.8288
22	50	8	42	0	855	0.8049	0.7866	0.8219
23	42	4	38	0	805	0.8009	0.7822	0.8182
24	71	8	63	0	763	0.7925	0.7731	0.8105

Table S12. 2021-2022 Cohort, prediabetes-patient classified as the Medium-risk subgroup.

	removed	observed	censored	entrance	at_risk	Medium-Risk	lower_0.95	upper_0.95
0	0	0	0	2412	2412	1	1	1
1	53	53	0	0	2412	0.9780	0.9713	0.9832
2	123	123	0	0	2359	0.9270	0.9159	0.9367
3	78	78	0	0	2236	0.8947	0.8818	0.9063
4	96	96	0	0	2158	0.8549	0.8402	0.8683
5	66	66	0	0	2062	0.8275	0.8119	0.8420
6	84	84	0	0	1996	0.7927	0.7760	0.8083
7	92	92	0	0	1912	0.7546	0.7369	0.7712
8	70	70	0	0	1820	0.7255	0.7073	0.7429
9	77	77	0	0	1750	0.6936	0.6748	0.7116
10	59	59	0	0	1673	0.6692	0.6500	0.6875
11	171	57	114	0	1614	0.6455	0.6261	0.6642
12	247	64	183	0	1443	0.6169	0.5971	0.6360
13	340	30	310	0	1196	0.6014	0.5813	0.6209
14	214	23	191	0	856	0.5853	0.5646	0.6053
15	40	11	29	0	642	0.5752	0.5541	0.5957
16	24	7	17	0	602	0.5685	0.5471	0.5894
17	31	11	20	0	578	0.5577	0.5357	0.5791
18	47	18	29	0	547	0.5394	0.5165	0.5616
19	34	11	23	0	500	0.5275	0.5041	0.5504
20	50	13	37	0	466	0.5128	0.4887	0.5363
21	38	14	24	0	416	0.4955	0.4706	0.5199
22	42	11	31	0	378	0.4811	0.4555	0.5063
23	30	5	25	0	336	0.4740	0.4479	0.4995
24	30	7	23	0	306	0.4631	0.4365	0.4893

8. KM-Survival Tables for PD onset HbA1c group's Risk Subgroups.

8.1 2018-2020 Cohort with 5.7-5.9 PD onset HbA1c across Risk Subgroups.

Table S12. PD onset patients with 5.7-5.9% HbA1c from 2018-2020 Cohort, prediabetes-patient classified as the high-risk subgroup

	removed	observed	censored	entrance	at_risk	High-Risk	lower_0.95	upper_0.95
0	0	0	0	1197	1197	1	1	1
1	138	138	0	0	1197	0.8847	0.8652	0.9015
2	160	160	0	0	1059	0.751	0.7255	0.7746
3	106	106	0	0	899	0.6625	0.6349	0.6885
4	127	127	0	0	793	0.5564	0.5278	0.584
5	96	96	0	0	666	0.4762	0.4476	0.5042
6	89	89	0	0	570	0.4018	0.374	0.4295
7	87	87	0	0	481	0.3292	0.3027	0.3559
8	85	85	0	0	394	0.2581	0.2337	0.2832
9	68	68	0	0	309	0.2013	0.1791	0.2245
10	65	65	0	0	241	0.147	0.1276	0.1677
11	59	57	2	0	176	0.0994	0.0833	0.1172
12	52	52	0	0	117	0.0552	0.0432	0.0692
13	20	17	3	0	65	0.0408	0.0306	0.0531
14	3	1	2	0	45	0.0399	0.0298	0.0521
15	4	4	0	0	42	0.0361	0.0265	0.0479
16	1	1	0	0	38	0.0351	0.0256	0.0468
17	1	1	0	0	37	0.0342	0.0248	0.0458
18	2	1	1	0	36	0.0332	0.024	0.0447
19	2	1	1	0	34	0.0323	0.0232	0.0436
20	1	0	1	0	32	0.0323	0.0232	0.0436
22	1	1	0	0	31	0.0312	0.0223	0.0425
23	1	1	0	0	30	0.0302	0.0214	0.0413
24	4	1	3	0	29	0.0291	0.0205	0.0402

Table S13: PD onset patients with 5.7-5.9% HbA1c from 2018-2020 Cohort, prediabetes-patient classified as the low-risk subgroup

	removed	observed	censored	entrance	at_risk	Low-Risk	lower_0.95	upper_0.95
0	0	0	0	3804	3804	1	1	1
1	4	4	0	0	3804	0.9989	0.9972	0.9996
2	8	8	0	0	3800	0.9968	0.9945	0.9982
3	3	3	0	0	3792	0.9961	0.9935	0.9976
4	5	5	0	0	3789	0.9947	0.9919	0.9966
5	5	5	0	0	3784	0.9934	0.9903	0.9956
6	3	3	0	0	3779	0.9926	0.9894	0.9949
7	6	6	0	0	3776	0.9911	0.9875	0.9936
8	3	3	0	0	3770	0.9903	0.9866	0.9929
9	3	3	0	0	3767	0.9895	0.9857	0.9923
10	4	4	0	0	3764	0.9884	0.9845	0.9914
11	68	7	61	0	3760	0.9866	0.9824	0.9898
12	107	4	103	0	3692	0.9855	0.9812	0.9889
13	303	26	277	0	3585	0.9784	0.9732	0.9826
14	190	17	173	0	3282	0.9733	0.9676	0.9781
15	48	9	39	0	3092	0.9705	0.9644	0.9755
16	14	7	7	0	3044	0.9682	0.9619	0.9735
17	31	15	16	0	3030	0.9635	0.9567	0.9692
18	32	12	20	0	2999	0.9596	0.9525	0.9657
19	40	14	26	0	2967	0.9551	0.9476	0.9615
20	26	11	15	0	2927	0.9515	0.9437	0.9582
21	20	11	9	0	2901	0.9479	0.9398	0.9549
22	35	10	25	0	2881	0.9446	0.9363	0.9518
23	19	8	11	0	2846	0.9419	0.9334	0.9494
24	31	10	21	0	2827	0.9386	0.9299	0.9463

Table S14: PD onset patients with 5.7-5.9% HbA1c from 2018-2020 Cohort, prediabetes-patient classified as the Medium-risk subgroup

	removed	observed	censored	entrance	at_risk	Medium-Risk	lower_0.95	upper_0.95
0	0	0	0	2627	2627	1	1	1
1	50	50	0	0	2627	0.9810	0.9750	0.9855
2	120	120	0	0	2577	0.9353	0.9252	0.9441
3	106	106	0	0	2457	0.8949	0.8826	0.9061
4	115	115	0	0	2351	0.8512	0.8370	0.8642
5	98	98	0	0	2236	0.8139	0.7984	0.8282
6	89	89	0	0	2138	0.7800	0.7636	0.7953
7	100	100	0	0	2049	0.7419	0.7247	0.7582
8	84	84	0	0	1949	0.7099	0.6922	0.7269
9	76	76	0	0	1865	0.6810	0.6628	0.6985
10	69	69	0	0	1789	0.6547	0.6362	0.6726
11	154	89	65	0	1720	0.6209	0.6020	0.6391
12	154	70	84	0	1566	0.5931	0.5740	0.6117
13	146	36	110	0	1412	0.5780	0.5587	0.5967
14	111	26	85	0	1266	0.5661	0.5467	0.5850
15	34	14	20	0	1155	0.5593	0.5398	0.5782
16	16	11	5	0	1121	0.5538	0.5342	0.5728
17	19	14	5	0	1105	0.5468	0.5271	0.5659
18	18	11	7	0	1086	0.5412	0.5215	0.5605
19	16	8	8	0	1068	0.5372	0.5174	0.5565
20	20	11	9	0	1052	0.5315	0.5117	0.5510
21	18	15	3	0	1032	0.5238	0.5039	0.5433
22	13	5	8	0	1014	0.5212	0.5013	0.5408
23	18	12	6	0	1001	0.5150	0.4950	0.5346
24	14	4	10	0	983	0.5129	0.4928	0.5326

8.2. 2018-2020 Cohort with 6.0-6.4 PD onset HbA1c across Risk Subgroups.

Table S15: 2018-2020 Cohort with 6.0-6.4 PD onset HbA1c, High Risk subgroups

	removed	observed	censored	entrance	at_risk	High-Risk	lower_0.95	upper_0.95
0	0	0	0	1197	1197	1	1	1
1	138	138	0	0	1197	0.8847	0.8652	0.9015
2	160	160	0	0	1059	0.751	0.7255	0.7746
3	106	106	0	0	899	0.6625	0.6349	0.6885
4	127	127	0	0	793	0.5564	0.5278	0.584
5	96	96	0	0	666	0.4762	0.4476	0.5042
6	89	89	0	0	570	0.4018	0.374	0.4295
7	87	87	0	0	481	0.3292	0.3027	0.3559
8	85	85	0	0	394	0.2581	0.2337	0.2832
9	68	68	0	0	309	0.2013	0.1791	0.2245
10	65	65	0	0	241	0.147	0.1276	0.1677
11	59	57	2	0	176	0.0994	0.0833	0.1172
12	52	52	0	0	117	0.0552	0.0432	0.0692
13	20	17	3	0	65	0.0408	0.0306	0.0531
14	3	1	2	0	45	0.0399	0.0298	0.0521
15	4	4	0	0	42	0.0361	0.0265	0.0479
16	1	1	0	0	38	0.0351	0.0256	0.0468
17	1	1	0	0	37	0.0342	0.0248	0.0458
18	2	1	1	0	36	0.0332	0.024	0.0447
19	2	1	1	0	34	0.0323	0.0232	0.0436
20	1	0	1	0	32	0.0323	0.0232	0.0436
22	1	1	0	0	31	0.0312	0.0223	0.0425
24	1	1	0	0	30	0.0302	0.0214	0.0413

Table S16: 2018-2020 Cohort with 6.0-6.4 PD onset HbA1c, Low Risk subgroups

	removed	observed	censored	entrance	at_risk	Low-Risk	lower_0.95	upper_0.95
0	0	0	0	3804	3804	1	1	1
1	4	4	0	0	3804	0.9989	0.9972	0.9996
2	8	8	0	0	3800	0.9968	0.9945	0.9982
3	3	3	0	0	3792	0.9961	0.9935	0.9976
4	5	5	0	0	3789	0.9947	0.9919	0.9966
5	5	5	0	0	3784	0.9934	0.9903	0.9956
6	3	3	0	0	3779	0.9926	0.9894	0.9949
7	6	6	0	0	3776	0.9911	0.9875	0.9936
8	3	3	0	0	3770	0.9903	0.9866	0.9929
9	3	3	0	0	3767	0.9895	0.9857	0.9923
10	4	4	0	0	3764	0.9884	0.9845	0.9914
11	68	7	61	0	3760	0.9866	0.9824	0.9898
12	107	4	103	0	3692	0.9855	0.9812	0.9889
13	303	26	277	0	3585	0.9784	0.9732	0.9826
14	190	17	173	0	3282	0.9733	0.9676	0.9781
15	48	9	39	0	3092	0.9705	0.9644	0.9755
16	14	7	7	0	3044	0.9682	0.9619	0.9735
17	31	15	16	0	3030	0.9635	0.9567	0.9692
18	32	12	20	0	2999	0.9596	0.9525	0.9657
19	40	14	26	0	2967	0.9551	0.9476	0.9615
20	26	11	15	0	2927	0.9515	0.9437	0.9582
21	20	11	9	0	2901	0.9479	0.9398	0.9549
22	35	10	25	0	2881	0.9446	0.9363	0.9518
23	19	8	11	0	2846	0.9419	0.9334	0.9494
24	31	10	21	0	2827	0.9386	0.9299	0.9463

Table S17: 2018-2020 Cohort with 6.0-6.4 PD onset HbA1c, Medium Risk subgroups

	removed	observed	censored	entrance	at_risk	Medium-Risk	lower_0.95	upper_0.95
0	0	0	0	2627	2627	1	1	1
1	50	50	0	0	2627	0.9810	0.9750	0.9855
2	120	120	0	0	2577	0.9353	0.9252	0.9441
3	106	106	0	0	2457	0.8949	0.8826	0.9061
4	115	115	0	0	2351	0.8512	0.8370	0.8642
5	98	98	0	0	2236	0.8139	0.7984	0.8282
6	89	89	0	0	2138	0.7800	0.7636	0.7953
7	100	100	0	0	2049	0.7419	0.7247	0.7582
8	84	84	0	0	1949	0.7099	0.6922	0.7269
9	76	76	0	0	1865	0.6810	0.6628	0.6985
10	69	69	0	0	1789	0.6547	0.6362	0.6726
11	154	89	65	0	1720	0.6209	0.6020	0.6391
12	154	70	84	0	1566	0.5931	0.5740	0.6117
13	146	36	110	0	1412	0.5780	0.5587	0.5967
14	111	26	85	0	1266	0.5661	0.5467	0.5850
15	34	14	20	0	1155	0.5593	0.5398	0.5782
16	16	11	5	0	1121	0.5538	0.5342	0.5728
17	19	14	5	0	1105	0.5468	0.5271	0.5659
18	18	11	7	0	1086	0.5412	0.5215	0.5605
19	16	8	8	0	1068	0.5372	0.5174	0.5565
20	20	11	9	0	1052	0.5315	0.5117	0.5510
21	18	15	3	0	1032	0.5238	0.5039	0.5433
22	13	5	8	0	1014	0.5212	0.5013	0.5408
23	18	12	6	0	1001	0.5150	0.4950	0.5346
24	14	4	10	0	983	0.5129	0.4928	0.5326

8.3. 2021-2022 Cohort with 5.7-5.9 PD onset HbA1c across Risk Subgroups.

Table S18. 2021-2022 Cohort with 5.7-5.9 PD onset HbA1c, High Risk subgroups

	removed	observed	censored	entrance	at_risk	High-Risk	lower_0.95	upper_0.95
0	0	0	0	719	719	1	1	1
1	72	72	0	0	719	0.8999	0.8755	0.9197
2	83	83	0	0	647	0.7844	0.7525	0.8127
3	62	62	0	0	564	0.6982	0.6632	0.7303
4	61	61	0	0	502	0.6134	0.5767	0.6479
5	55	55	0	0	441	0.5369	0.4997	0.5725
6	32	32	0	0	386	0.4924	0.4553	0.5283
7	36	36	0	0	354	0.4423	0.4057	0.4782
8	34	34	0	0	318	0.3950	0.3592	0.4306
9	30	30	0	0	284	0.3533	0.3185	0.3882
10	26	26	0	0	254	0.3171	0.2834	0.3513
11	36	21	15	0	228	0.2879	0.2552	0.3213
12	58	31	27	0	192	0.2414	0.2105	0.2735
13	39	11	28	0	134	0.2216	0.1913	0.2533
14	36	10	26	0	95	0.1983	0.1683	0.2301
15	8	3	5	0	59	0.1882	0.1579	0.2206
16	1	1	0	0	51	0.1845	0.1540	0.2172
17	2	1	1	0	50	0.1808	0.1502	0.2137
18	6	5	1	0	48	0.1620	0.1310	0.1959
19	1	0	1	0	42	0.1620	0.1310	0.1959
20	1	0	1	0	41	0.1620	0.1310	0.1959
21	2	0	2	0	40	0.1620	0.1310	0.1959
22	6	4	2	0	38	0.1449	0.1137	0.1798
23	3	1	2	0	32	0.1404	0.1092	0.1755
24	3	1	2	0	29	0.1356	0.1043	0.1709

Table S19. 2021-2022 Cohort with 5.7-5.9 PD onset HbA1c, low Risk subgroups

	removed	observed	censored	entrance	at_risk	Low-Risk	lower_0.95	upper_0.95
0	0	0	0	3677	3677	1	1	1
1	2	2	0	0	3677	0.9995	0.9978	0.9999
2	35	35	0	0	3675	0.9899	0.9861	0.9927
3	33	33	0	0	3640	0.9810	0.9760	0.9849
4	36	36	0	0	3607	0.9712	0.9652	0.9761
5	32	32	0	0	3571	0.9625	0.9558	0.9681
6	25	25	0	0	3539	0.9557	0.9485	0.9619
7	33	33	0	0	3514	0.9467	0.9389	0.9535
8	36	36	0	0	3481	0.9369	0.9286	0.9443
9	32	32	0	0	3445	0.9282	0.9194	0.9361
10	26	26	0	0	3413	0.9211	0.9119	0.9294
11	197	32	165	0	3387	0.9124	0.9028	0.9211
12	319	21	298	0	3190	0.9064	0.8965	0.9154
13	975	37	938	0	2871	0.8947	0.8842	0.9043
14	532	27	505	0	1896	0.8820	0.8705	0.8925
15	109	16	93	0	1364	0.8717	0.8592	0.8831
16	33	10	23	0	1255	0.8647	0.8515	0.8768
17	50	15	35	0	1222	0.8541	0.8399	0.8671
18	69	14	55	0	1172	0.8439	0.8289	0.8577
19	89	11	78	0	1103	0.8355	0.8197	0.8500
20	82	15	67	0	1014	0.8231	0.8063	0.8386
21	77	12	65	0	932	0.8125	0.7948	0.8288
22	50	8	42	0	855	0.8049	0.7866	0.8219
23	42	4	38	0	805	0.8009	0.7822	0.8182
24	71	8	63	0	763	0.7925	0.7731	0.8105

Table S20. 2021-2022 Cohort with 5.7-5.9 PD onset HbA1c, Medium Risk subgroups

	removed	observed	censored	entrance	at_risk	Medium-Risk	Medium-Risk_lower_0.95	Medium-Risk_upper_0.95
0	0	0	0	2412	2412	1	1	1
1	53	53	0	0	2412	0.9780	0.9713	0.9832
2	123	123	0	0	2359	0.9270	0.9159	0.9367
3	78	78	0	0	2236	0.8947	0.8818	0.9063
4	96	96	0	0	2158	0.8549	0.8402	0.8683
5	66	66	0	0	2062	0.8275	0.8119	0.8420
6	84	84	0	0	1996	0.7927	0.7760	0.8083
7	92	92	0	0	1912	0.7546	0.7369	0.7712
8	70	70	0	0	1820	0.7255	0.7073	0.7429
9	77	77	0	0	1750	0.6936	0.6748	0.7116
10	59	59	0	0	1673	0.6692	0.6500	0.6875
11	171	57	114	0	1614	0.6455	0.6261	0.6642
12	247	64	183	0	1443	0.6169	0.5971	0.6360
13	340	30	310	0	1196	0.6014	0.5813	0.6209
14	214	23	191	0	856	0.5853	0.5646	0.6053
15	40	11	29	0	642	0.5752	0.5541	0.5957
16	24	7	17	0	602	0.5685	0.5471	0.5894
17	31	11	20	0	578	0.5577	0.5357	0.5791
18	47	18	29	0	547	0.5394	0.5165	0.5616
19	34	11	23	0	500	0.5275	0.5041	0.5504
20	50	13	37	0	466	0.5128	0.4887	0.5363
21	38	14	24	0	416	0.4955	0.4706	0.5199
22	42	11	31	0	378	0.4811	0.4555	0.5063
23	30	5	25	0	336	0.4740	0.4479	0.4995
24	30	7	23	0	306	0.4631	0.4365	0.4893

8.4. 2021-2022 Cohort with 6.0-6.4 PD onset HbA1c across Risk Subgroups.

Table S21. 2021-2022 Cohort with 6.0-6.4 PD onset HbA1c, High Risk subgroups

	removed	observed	censored	entrance	at_risk	High-Risk	lower_0.95	upper_0.95
0	0	0	0	719	719	1	1	1
1	72	72	0	0	719	0.8999	0.8755	0.9197
2	83	83	0	0	647	0.7844	0.7525	0.8127
3	62	62	0	0	564	0.6982	0.6632	0.7303
4	61	61	0	0	502	0.6134	0.5767	0.6479
5	55	55	0	0	441	0.5369	0.4997	0.5725
6	32	32	0	0	386	0.4924	0.4553	0.5283
7	36	36	0	0	354	0.4423	0.4057	0.4782
8	34	34	0	0	318	0.3950	0.3592	0.4306
9	30	30	0	0	284	0.3533	0.3185	0.3882
10	26	26	0	0	254	0.3171	0.2834	0.3513
11	36	21	15	0	228	0.2879	0.2552	0.3213
12	58	31	27	0	192	0.2414	0.2105	0.2735
13	39	11	28	0	134	0.2216	0.1913	0.2533
14	36	10	26	0	95	0.1983	0.1683	0.2301
15	8	3	5	0	59	0.1882	0.1579	0.2206
16	1	1	0	0	51	0.1845	0.1540	0.2172
17	2	1	1	0	50	0.1808	0.1502	0.2137
18	6	5	1	0	48	0.1620	0.1310	0.1959
19	1	0	1	0	42	0.1620	0.1310	0.1959
20	1	0	1	0	41	0.1620	0.1310	0.1959
21	2	0	2	0	40	0.1620	0.1310	0.1959
22	6	4	2	0	38	0.1449	0.1137	0.1798
23	3	1	2	0	32	0.1404	0.1092	0.1755
24	3	1	2	0	29	0.1356	0.1043	0.1709

Table S22. 2021-2022 Cohort with 6.0-6.4 PD onset HbA1c, Low Risk subgroups

	removed	observed	censored	entrance	at_risk	Low-Risk	lower_0.95	upper_0.95
0	0	0	0	3677	3677	1	1	1
1	2	2	0	0	3677	0.9995	0.9978	0.9999
2	35	35	0	0	3675	0.9899	0.9861	0.9927
3	33	33	0	0	3640	0.9810	0.9760	0.9849
4	36	36	0	0	3607	0.9712	0.9652	0.9761
5	32	32	0	0	3571	0.9625	0.9558	0.9681
6	25	25	0	0	3539	0.9557	0.9485	0.9619
7	33	33	0	0	3514	0.9467	0.9389	0.9535
8	36	36	0	0	3481	0.9369	0.9286	0.9443
9	32	32	0	0	3445	0.9282	0.9194	0.9361
10	26	26	0	0	3413	0.9211	0.9119	0.9294
11	197	32	165	0	3387	0.9124	0.9028	0.9211
12	319	21	298	0	3190	0.9064	0.8965	0.9154
13	975	37	938	0	2871	0.8947	0.8842	0.9043
14	532	27	505	0	1896	0.8820	0.8705	0.8925
15	109	16	93	0	1364	0.8717	0.8592	0.8831
16	33	10	23	0	1255	0.8647	0.8515	0.8768
17	50	15	35	0	1222	0.8541	0.8399	0.8671
18	69	14	55	0	1172	0.8439	0.8289	0.8577
19	89	11	78	0	1103	0.8355	0.8197	0.8500
20	82	15	67	0	1014	0.8231	0.8063	0.8386
21	77	12	65	0	932	0.8125	0.7948	0.8288
22	50	8	42	0	855	0.8049	0.7866	0.8219
23	42	4	38	0	805	0.8009	0.7822	0.8182
24	71	8	63	0	763	0.7925	0.7731	0.8105

Table S23. 2021-2022 Cohort with 6.0-6.4 PD onset HbA1c, Medium Risk subgroups

	removed	observed	censored	entrance	at_risk	Medium-Risk	lower_0.95	upper_0.95
0	0	0	0	2412	2412	1	1	1
1	53	53	0	0	2412	0.9780	0.9713	0.9832
2	123	123	0	0	2359	0.9270	0.9159	0.9367
3	78	78	0	0	2236	0.8947	0.8818	0.9063
4	96	96	0	0	2158	0.8549	0.8402	0.8683
5	66	66	0	0	2062	0.8275	0.8119	0.8420
6	84	84	0	0	1996	0.7927	0.7760	0.8083
7	92	92	0	0	1912	0.7546	0.7369	0.7712
8	70	70	0	0	1820	0.7255	0.7073	0.7429
9	77	77	0	0	1750	0.6936	0.6748	0.7116
10	59	59	0	0	1673	0.6692	0.6500	0.6875
11	171	57	114	0	1614	0.6455	0.6261	0.6642
12	247	64	183	0	1443	0.6169	0.5971	0.6360
13	340	30	310	0	1196	0.6014	0.5813	0.6209
14	214	23	191	0	856	0.5853	0.5646	0.6053
15	40	11	29	0	642	0.5752	0.5541	0.5957
16	24	7	17	0	602	0.5685	0.5471	0.5894
17	31	11	20	0	578	0.5577	0.5357	0.5791
18	47	18	29	0	547	0.5394	0.5165	0.5616
19	34	11	23	0	500	0.5275	0.5041	0.5504
20	50	13	37	0	466	0.5128	0.4887	0.5363
21	38	14	24	0	416	0.4955	0.4706	0.5199
22	42	11	31	0	378	0.4811	0.4555	0.5063
23	30	5	25	0	336	0.4740	0.4479	0.4995
24	30	7	23	0	306	0.4631	0.4365	0.4893

Reference

1. Nicolaisen SK, Thomsen RW, Lau CJ, Sørensen HT, Pedersen L. Development of a 5-year risk prediction model for type 2 diabetes in individuals with incident HbA1c-defined pre-diabetes in Denmark. *BMJ Open Diabetes Research and Care*. 2022;10(5):e002946. doi:10.1136/bmjdrc-2022-002946

2. Liu Q, Zhou Q, He Y, Zou J, Guo Y, Yan Y. Predicting the 2-Year Risk of Progression from Prediabetes to Diabetes Using Machine Learning among Chinese Elderly Adults. *Journal of Personalized Medicine*. 2022;12(7):1055. doi:10.3390/jpm12071055

3. Han Y, Hu H, Liu Y, Wang Z, Liu D. Nomogram model and risk score to predict 5-year risk of progression from prediabetes to diabetes in Chinese adults: Development and validation of a novel model. *Diabetes, Obesity and Metabolism*. 2023;25(3):675-687. doi:10.1111/dom.14910

4. Aoki J, Khalid O, Kaya C, Nagymanyoki Z, Hussong J, Salama ME. Progression from Prediabetes to Diabetes in a Diverse U.S. Population: A Machine Learning Model. *Diabetes Technology & Therapeutics*. 2024;26(10):748-753. doi:10.1089/dia.2024.0052

5. Cahn A, Shoshan A, Sagiv T, et al. Prediction of progression from pre-diabetes to diabetes: Development and validation of a machine learning model. *Diabetes/Metabolism Research and Reviews*. 2020;36(2):e3252. doi:10.1002/dmrr.3252

6. Zueger T, Schallmoser S, Kraus M, Saar-Tsechansky M, Feuerriegel S, Stettler C. Machine Learning for Predicting the Risk of Transition from Prediabetes to Diabetes. *Diabetes Technology & Therapeutics*. 2022;24(11):842-847. doi:10.1089/dia.2022.0210

7. Ahlqvist E, Storm P, Käräjämäki A, et al. Novel subgroups of adult-onset diabetes and their association with outcomes: a data-driven cluster analysis of six variables. *The Lancet Diabetes & Endocrinology*. 2018;6(5):361-369. doi:10.1016/S2213-8587(18)30051-2

8. Chen T, Guestrin C. XGBoost: A Scalable Tree Boosting System. In: *Proceedings of the 22nd ACM SIGKDD International Conference on Knowledge Discovery and Data Mining*. KDD '16. Association for Computing Machinery; 2016:785-794. doi:10.1145/2939672.2939785
